# ATF3 aggravates kidney fibrosis via HDAC6-dependent epigenetic reprogramming

**DOI:** 10.7150/ijbs.125062

**Published:** 2026-01-01

**Authors:** Sibei Tao, Chenzhou Wu, Fanyuan Yu, Lina Yang, Lingzhi Li, Fan Guo, Ting Xiang, Liang Ma, Ping Fu

**Affiliations:** 1 Division of Nephrology, Kidney Research Institute, West China Hospital, Sichuan University, Chengdu 610041, Sichuan, China.; 2 State Key Laboratory of Oral Diseases & National Center for Stomatology & National Clinical Research Center for Oral Diseases, West China Hospital of Stomatology, Sichuan University, Chengdu 610041, Sichuan, China.

**Keywords:** kidney fibrosis, ATF3, HDAC6, epigenetic reprogramming

## Abstract

Kidney fibrosis is the most common pathology and endpoint of CKD. Unraveling the mechanisms of kidney fibrosis is crucial. Activating transcription factors (ATFs) are implicated in a range of kidney diseases, but their roles in kidney fibrosis remain underexplored. In our investigation, employing an unbiased screening of ATF expression in fibrotic kidneys via analyzing single-cell and bulk RNA sequencing, we identified that ATF3 as the key player, markedly upregulated in damaged tubular epithelial cells (TECs). Crucially, ATF3 deletion in mice markedly attenuated kidney fibrosis and abrogated fibrotic traits in injured TECs. At the molecular level, ATF3 was found to recruit HDAC6 to the *SMAD7* promoter, eradicating histone 3 lysine 14 acetylation (H3K14ac) and diminishing *SMAD7* transcription. This interaction between ATF3 and HDAC6 culminated in the suppression of *Smad7*, triggering the TGF-β/Smad3 pathway and exacerbating kidney fibrosis. Collectively, our findings shed light on the complex underpinnings of kidney fibrosis and herald novel therapeutic targets for combating CKD.

## Introduction

Approximately 850 million patients worldwide suffer from chronic kidney disease (CKD), a syndrome characterized by the persistent and irreversible loss of kidney function 1. CKD has become a global health crisis with increasing prevalence as well as high economic costs 2, 3. Kidney fibrosis is the most common pathology and endpoint of CKD, resulting in end-stage kidney disease (ESKD) 4, 5. Non-resolving inflammation is a major driving force in kidney fibrogenesis 6, 7, where multiple types of cells are involved in promoting scar formation and the progression of kidney fibrosis 8-11. Unfortunately, therapeutic options for kidney fibrosis are lacking 12 due to the limited understanding of its pathogenesis. Thus, it is urgent to explore the mechanisms underlying kidney fibrosis for effective treatments.

Activating transcription factors (ATFs) are a group of basic leucine zipper (bZIP) transcription factors with numerous physiological functions 13. Due to their extensive functions, ATFs have gained importance in many popular fields 14. Increasing evidence has shown that ATFs are involved in various kidney diseases and expressed in various types of cells in response to different stimuli 15-20. For example, ATF1 is activated by uremic toxins in endothelial cells 19. ATF2 is upregulated in small cysts in autosomal dominant polycystic kidney disease 16, and ATF3 is elevated in acute kidney injury (AKI) 17. ATF5 is induced in diabetic kidney disease 18. Furthermore, ATF4 and ATF6, which are stimulated during endoplasmic reticulum stress 21, 22, are also involved in various kidney diseases 15, 20, 23, 24. However, the role of ATFs in kidney fibrosis remains poorly understood.

ATFs recognize and combine with cAMP response element-binding proteins (CREB) for regulation of transcription 25. Additionally, recent studies have shown that ATFs can regulate transcription via epigenetic regulation through recruitment of cofactors 26, 27. The interaction between ATFs and histone deacetylases (HDACs) is shown to be key for inflammation resolution via regulation of transcriptional activity 27. In the ATF-HDAC complex, ATFs respond to stress stimuli and recruit HDACs to specific chromatin regions for transcriptional regulation. Limited evidence suggests that some ATF-HDAC interactions, including the ATF3-HDAC1 complex, can inhibit inflammatory responses during AKI 17 and protect the heart from hypertensive stimuli-induced cardiac fibrosis 28. However, the role of these interactions between ATFs and HDACs in kidney fibrosis remains largely unknown.

In this study, we used a stepwise strategy trying to discover the role and underlying mechanism of the master ATF responsible for fibrotic remodeling in the kidney. We first used a non-biased approach to identify increased ATFs expressions in fibrotic kidneys based on single-cell and bulk RNA sequencing data, and discovered ATF3 as a critical player. ATF3 knockout mice were used to further elucidate its role in kidney fibrosis. Subsequent mechanistic investigations revealed that ATF3 recruited HDAC6 to the *SMAD7* promoter region to erase H3K14 acetylation and suppress *SMAD7* transcriptional activity. The ATF3-HDAC6 interaction mediated *Smad7* suppression finally resulted in activated TGF-β/Smad3 signaling and accelerated the fibrotic process in the kidney.

## Materials and Methods

### Animal experiments

All experimental protocols for animal studies were approved by the Animal Care and Use Ethics Committee of Sichuan University (Approval No.2021296A). Mice were maintained on a 12-h light, 12-h dark cycle and on a standard chow diet. All mice were randomized allocated to the different groups (n=6 for each group) and investigators were blinded to analyses. Littermate control mice were used for all *in vivo* experiments. ATF3 global-knockout C57BL/6 mice (aged 8-10 weeks, weighted 25-28 g) were obtained from GemPharmatech (Nanjing, China). Age-matched littermates were used in all the experiments. All animals were randomly grouped (n = 6 mice per group), and the sexes were equally distributed. All mice were genotyped using PCR prior to the experiments. Unilateral ureteral obstruction (UUO) was induced in isoflurane-anesthetized mice by making an incision on the back to expose the left kidney. The urethra was ligated near the kidney, with similar ligation sites in all the animals. The mice were then sutured and allowed to recover in their home cages. The Sham group underwent a sham operation without urethral ligation. Mice were sacrificed at 7 days after UUO surgery. In the folic acid (FA) model, mice were intraperitoneally injected with 250 mg/kg FA (diluted in 0.3 M sodium bicarbonate) once, while the control mice were given the quivalent volume of solvent intraperitoneally. Mice were sacrificed 7 days after FA injection. Adenine group mice were fed 0.2% adenine mixed with AIN-93M diet (M21101502, MolDiets) for 2 weeks, while the control group were fed the normal diet. In the cisplatin-CKD model, mice received 7 mg/kg cisplatin (15663-27-1, Synguider, Chengdu, China) or saline vehicle via intraperitoneal injection once a week for 4 times. Mice were sacrificed 3 days after the final injection. All serum and kidney samples were collected for further analyses.

### Human kidney biopsy samples

Human kidney sections (3 μm) were obtained from renal biopsies performed at West China Hospital, Sichuan University, Chengdu, China. Studies involving human tissues were conducted with informed consent and approval from the Institutional Ethics Committee of West China Hospital, Sichuan University. Renal biopsies from patients had been performed as part of routine clinical diagnostic investigation and collected as Table S3. The samples of renal biopsies were obtained from Department of Pathology, West China hospital, Sichuan University. Normal control samples were obtained from the healthy kidney poles of individuals who underwent tumor nephrectomies without diabetes or other kidney diseases. The investigations were conducted in accordance with the principles of the Declaration of Helsinki and were approved by the Ethics Committee of Sichuan University after informed consents were obtained from the subjects.

### Cell culture

Mouse primary kidney tubular epithelial cells were isolated from 3-5 week-old mice. Mouse kidney epithelial cell line (TCMK-1), HEK 293T cells and rat renal interstitial fibroblasts (NRK-49F) cells were purchased from the American Type Culture Collection (Manassas, VA) and were cultured in DMEM (Sigma-Aldrich) containing 10% fetal bovine serum (FBS), at 37℃, 5% CO2, 95% air. Cells were starved with 0.5% FBS (SH30084.03, Hyclone, Beijing, China) for 24 h, then further treated with 20 ng/mL of TGF-β1 (R&D Systems, 240-B-002/CF, Minneapolis, MN, USA).

### Tail vein injection of adeno-associated virus (AAV)

Mice were fixed with a fixator to expose the tails. 100 µl filter-purified AAV2/9-Ksp-cadherin-m-Hdac6-3×flag-Null (1.2×10^12^ vg/mL) or AAV2/9-Ksp-cadherin-Null (1.6×10^12^ vg/mL) were delivered to the kidney by means of tail vein injection. No toxicity was observed in AAV-treated mice. Before 3 weeks of UUO surgery, adeno-associated virus vectors HBAAV2/9 targeting epithelial marker cadherin, harboring Hdac6 (HBAAV2/9-Ksp-cadherin-m-Hdac6-3×flag-Null, Hanheng Biotechnology Co., Ltd., Shanghai, China) or their negative controls (HBAAV2/9-Ksp-cadherin-Null) were injected through tail vein.

### Single-cell sequencing data processing

Single-cell RNA sequencing (scRNA-seq) data were obtained from the GEO database (GSE190887) and processed using the Seurat package in R software (v4.4.0). Pre-processed data were read from a serialized Seurat object. Gene expression normalization was performed using the NormalizeData function, followed by identification of highly variable genes and data scaling. Principal component analysis (PCA) was carried out using the top 50 principal components. To correct for batch effects across samples, the Harmony algorithm was employed with sample identity as the integration factor.

A range of dimensionalities (15-40) was tested to determine optimal resolution for clustering and UMAP visualization. Based on the resulting embeddings, 30 dimensions were selected for downstream analysis. Duplicate cells were removed from the Harmony embeddings prior to final dimensionality reduction. Uniform Manifold Approximation and Projection (UMAP) was performed using 30 Harmony dimensions with customized parameters (min. dist = 0.001, spread = 3, n. neighbors = 20), and clustering was conducted using the Louvain algorithm.

Cell type annotation was guided by existing metadata, and the identified clusters were visualized using UMAP plot with custom color palettes.

### RNA-seq

Total tissue RNA from kidneys (n = 3 for each group) was isolated using Trizol reagent (Invitrogen, Carlsbad, CA, USA). Sequencing library preparation and RNA sequencing were conducted at LC-Bio Technology Co., Ltd (Hangzhou, China). The libraries were sequenced on an Illumina NovaSeq^TM^ 6000 platform and 2×150-bp paired-end reads were generated. Bioinformatic analysis was further performed using the OmicStudio tools at https://www.omicstudio.cn/tool.

### Plasmids and siRNAs transfection

Plasmids and siRNAs were transfected using Endofectin^TM^ Max transfection reagent (EF014, GeneCopoeia, Rockville, USA) in accordance with its instructions for use. Plasmids were purchased from Wuhan Miaoling Biotechnology Co., Ltd (pEnCMV-ATF3 (human)-3×FLAG NM_001674.4, pCMV-ATF3(mouse)-3×Myc-SV40-Neo NM_007498.3, pGL3-SMAD7 (human)-Promoter-Fluc P33785, pGL3-Basic, pCMV-MCS-3×FLAG-Neo). The siRNA sequences are listed herein: HDAC6 (human) siRNA, sense 5'-CCAUGCCAUCAAGGAGCAATT-3', and antisense 5'-UUGCUCCUUGAUGGCAUGGTT-3', HDAC6 (mouse) siRNA, sense 5'-GUGGCCGUAUUAUUCUUAUTT-3', and antisense 5'-AUAAGAAUAAUACGGCCACTT-3'. TCMK-1 or 293T cells, seeded in six-well plates, were transfected with 1 μg plasmids with/without a final concentration of 200 nM HDAC6 or scramble siRNAs, using 5 μL Endofectin reagent dissolved in 100 μL optiMEM, added in 1 mL DMEM containing 10% FBS for 24 h, and then 1 mL DMEM containing 10% FBS was added for another 24 h incubation. In the meantime, the cells were treated with/without TGF-β1 (20 ng/mL). The plasmid sequence for pGL3-SMAD7 (human)-Promoter-Fluc is listed on: http://www.miaolingbio.com/plasmid/P33785.html. Sequences were designed based on the NCBI database (NC_000018.10).

### Enzyme linked immunosorbent assay (ELISA)

Cell culture supernatant TGF-β1 concentrations were tested by using ELISA kits purchased from Quanzhou Ruixin Biotech Co., Ltd (China). The specific operation procedures were following manufacturers' instructions.

### Extraction and culture of primary renal tubular epithelial cells

Briefly, PTC were isolated from collagenase-digested kidneys obtained from each group of C57BL/6 mice (3-4 weeks old). The kidneys were removed, minced into 1-mm pieces, and digested in collagenase (17100-017, Thermo Fisher Scientific, Waltham, MA, USA) at 37 °C for 30 min. After digestion, the supernatant was passed through three nylon sieves (pore sizes of 100, 70, and 40 μm). The proximal tubules retained on the 40 μm sieve were resuspended in RPMI 1640 (SH30027.LS, Hyclone, Beijing, China) with 10% FBS. After centrifuging for 10 min at 1000 rpm, the supernatant was discarded, and 1 ml erythrocyte lysate (R1010, Solarbio, Beijing, China) were added to remove red blood cells for 2-3 min on ice. The solution was then centrifuged for 5 min at 1000 rpm, 4 ℃. Finally, the supernatant was discarded and resuspended in appropriate amount of culture medium: RPMI 1640 with 10% FBS, 1% penicillin-streptomycin solution (SV30010, HyClone, Beijing, China), 1% insulin-transferrin-selenium (abs9463, Absin, Shanghai, China), and 20 ng/mL epidermal growth factor (EGF) (RP-10914, Invitrogen, CA, USA) at 37 °C in a humidified atmosphere of 5% CO_2_ and 95% air. The medium was replaced every two days. PTCs were starved with 0.5% FBS for 24 h, and further treated with 20 ng/mL of TGF-β1.

### Histological evaluation

Formalin-fixed, paraffin-embedded mouse kidney tissues were sectioned at a thickness of 4 μm. The sections were deparaffinized, rehydrated and stained with hematoxylin-eosin (HE) or/and Masson's trichrome (MASSON). The criteria for histological analysis were the same as described previously 29. ImageJ program (National Institutes of Health, Bethesda, MD, USA) was used.

### Immunohistochemistry

Kidney tissues were fixed in formalin, paraffin embedded, deparaffinized, rehydrated, and antigen retrieved. Slides were blocked with 2.5% normal goat serum, and incubated with primary antibodies at 4 °C overnight. The slides were washed three times in phosphate-buffered saline (PBS) and stained using VECTASTAIN ABC Kit (Vector, Burlingame, CA, USA). Images were captured using an AxioCamHRc digital camera (Carl Zeiss, Jena, Germany) at ×200 and ×400 magnifications with ZEN 2012 microscopy software. The proportion of positive area was calculated at ×200 manifestations with ImageJ software (version 1.52a; National Institutes of Health, Bethesda, MD).

### Immunofluorescence staining

Deparaffinized sections (4 μm) were incubated with PBS containing 5% horse serum for 1 h at room temperature to block non-specific binding sites. Then the specimens were incubated with primary antibodies in a humidified chamber overnight at 4 °C. Subsequently after washing, secondary antibody (1:500, Jackson ImmunoResearch, West Grove, PA, USA) was employed for 1 h. Nuclei were stained with DAPI (1:500, D8200, Solarbio, Beijing, China) during the final wash, and coverslips were mounted. Images were acquired by Stellaris confocal microscopy system (Leica, Wetzlar, Germany) at ×400 magnification.

### Western blot analysis and quantitative real-time PCR analysis

Western blot analysis and Quantitative real-time PCR analysis were performed as previously described 30. GAPDH was used as a loading control in western blot analysis. In the quantitative real-time PCR analysis, the relative gene quantities were calculated by the 2^-∆∆Ct^ method in comparison with the expression levels of GAPDH. Antibodies and primer sources are provided in Table S2 and S3.

### Co-IP

HEK 293T cells, seeded in six-well plates, were treated with TGF-β1 (20 ng/mL) in DMEM containing 10% FBS for 24 h, and then lysed by IP buffer (P0013, Beyotime, Shanghai, China), incubated with anti-ATF3 antibody overnight at 4 °C. Protein A/G magnetic beads (B23202, Bimake, Shanghai, China) were subsequently added and the mixture were incubated at 4 °C for 1 h. The beads were collected by magnetic separation and the non-binding supernatant was discarded. Bound protein was eluted by boiling at 100 °C for 5 min. Samples were subjected to western blotting to evaluate the interaction of ATF3 and HDAC6.

### ChIP

293T cells were starved with 0.5% FBS for 24 h, and further treated with 20 ng/mL of TGF-β1 for anti-ATF3 ChIP. Cellular chromatin was cross-linked by adding 1% formaldehyde for 3 minutes. The cross-linking was terminated with the addition of 125 mM glycine. The cells were washed twice by cold PBS, then resolved in a buffer containing 1 mM phenylmethanesulfonyl fluoride, 10 mM Tris-HCl (pH 8.0), 1% Triton X-100 and protease inhibitor cocktail, and 1% sodium deoxycholate for 10 min at 4 °C. Sonication was conducted for shearing chromatin into around 200-bp to 500-bp fragments. The supernatant was collected after centrifugation (12,000 g for 3 min at 4 °C) and equally divided into three tubes. Antibodies containing anti-ATF3 or anti-H3K14ac or anti-IgG obtained from Abcam were added into each tube and incubated at 4 ℃ for 3 h. Protein A/G magnetic beads (B23202, Bimake, Shanghai, China) linked with protein and antibodies were used for immunoprecipitation. The purified DNA was analyzed by quantitative real-time PCR. The primers used for ChIP-qPCR assay are listed in Table S4.

### Statistical analysis

Data are provided as mean ± standard deviation (SD). Statistical analysis for data with a normal distribution among multiple groups were determined using one-way analysis of variance (ANOVA), and pairwise comparisons were performed using the SNK test. All statistical analyses were conducted using the SPSS 21.0. Statistical significance was set at P < 0.05.

## Results

### Identification and characterization of ATF3 in kidney fibrosis

**Figure 1:**
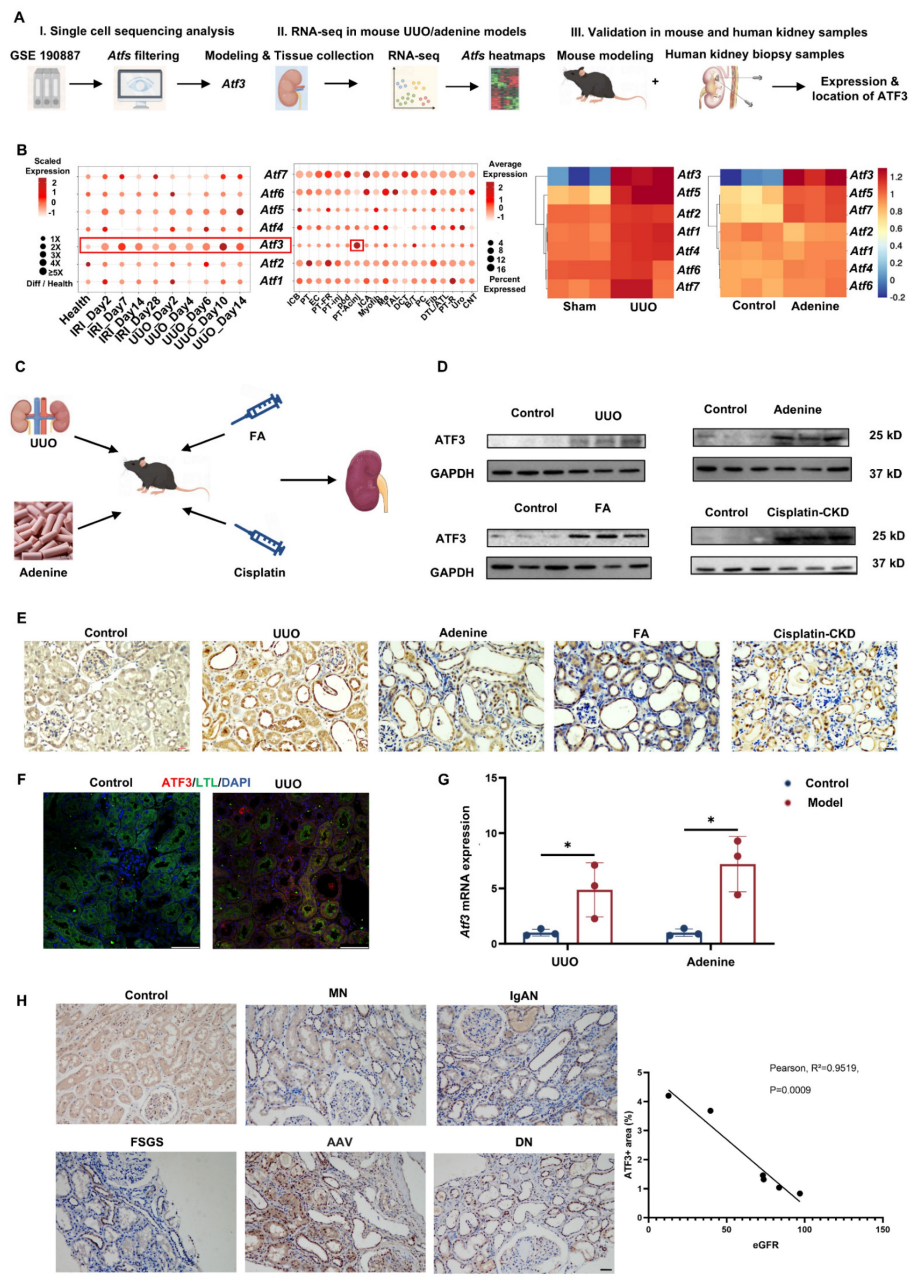
** ATF3 as a major responding ATF in kidney fibrosis.** (A) Flowchart of this part of experiment: I. Single cell sequencing analysis, II. RNA-seq in mouse UUO/adenine models, III. Validation in mouse and human kidney samples. (B) Left: scRNA-seq data analysis showing *Atf3* changes and enrichment in IRI/UUO mouse kidneys. PT-Acinj, Proximal tubular cells-Acute injury; PT-inj, Proximal tubular cells-injury; PT-R, Proximal tubular cells- Recovering. Right: heatmap for RNA-seq results for *Atf*s in UUO and adenine-induced fibrosis models. (C) Mouse modeling diagram. (D) Western blotting of ATF3 in UUO, adenine, FA and cisplatin-CKD mouse kidneys and relative controls. (E) Immunochemistry staining (×400, scale bar = 20 μm) of ATF3 in UUO, adenine, FA and cisplatin-CKD mouse kidneys and relative controls. (F) Immunofluorescence (×400, scale bar = 50 μm) of ATF3 double-stained by ATF3, LTL, DAPI, respectively, in UUO and control kidneys. (G) Quantitative real-time PCR analysis of ATF3 in UUO, adenine kidneys. (H) Immunochemistry staining (×200, scale bar = 50 μm) of ATF3 in MN (membranous nephropathy), IgAN (IgA nephropathy), FSGS (focal segmental glomerulosclerosis), AAV (anti-neutrophil cytoplasmic antibodies-associated vasculitis) and DN (diabetic nephropathy) patients' kidneys and adjacent-renal carcinoma normal kidney tissues, and the correlation analysis of IHC scores of ATF3 with eGFR. *p<0.05.

To identify the major ATF(s) participating in kidney fibrosis, we first analyzed the expression of ATFs in fibrotic kidneys (Fig. 1A). Single-cell RNA sequencing (scRNA-seq) data obtaining from the GEO database (GSE190887) 31,32 indicated that in ischemia/reperfusion injury (IRI) and unilateral ureter obstruction (UUO) models, transcription levels of *Atf3* increased most significantly among all the ATFs, in comparison to the controls (Fig. 1B, Supplementary Fig. S1A). More importantly, in contrast to other *Atf*s, which are ubiquitously expressed in all cell subtypes in injured kidneys, *Atf3* was expressed mostly in proximal tubular epithelial cells (Fig. 1B, left). This expression pattern indicates that *Atf3* plays a potential critical role in the parenchymal cells of kidney suffering acute injuries.

This was further confirmed by bulk RNA-sequencing of kidneys from UUO and adenine-induced nephrotoxicity mouse models (Fig. 1A). Akin to the previous data, *Atf3* was the most significantly upregulated ATFs during kidney fibrosis (Fig. 1B, right). Real-time qPCR findings for *Atf3* were consistent with the RNA-seq results (Fig. 1G) while it was consistently elevated at the protein level in UUO, adenine, folic acid (FA), and cisplatin-induced CKD in mouse kidneys (Fig. 1C-D).

The kidney tissue atlas from the Kidney Precision Medicine Project 33 also showed that *Atf3* expression was mostly enriched in the PTs of patients with CKD (Supplementary Fig. S1B). Immunochemical (IHC) staining showed that elevated ATF3 expression was mainly localized in the tubular epithelial cells (TECs) of UUO, adenine, FA, and cisplatin-CKD models (Fig. 1E, Supplementary Fig. S1C). Furthermore, immunofluorescence results confirmed an elevation of ATF3 expression in TEC in UUO mice model by dual staining for ATF3 and the proximal tubular epithelial cell marker LTL (Fig. 1F). In contrast, we also found that fibroblasts (α-SMA) and macrophages (F4/80) from UUO kidneys were negative for ATF3 immunostaining (Supplementary Fig. S1D).

Next, we validated upregulation of ATF3 in renal biopsies from patients with different fibrotic renal diseases, including membranous nephropathy, IgA nephropathy, focal segmental glomerulosclerosis, anti-neutrophil cytoplasmic antibodies-associated vasculitis, and diabetic nephropathy, in comparison to normal kidney tissues by IHC (Fig. 1A). Notably, the immunohistochemical scores for ATF3 negatively correlated with estimated glomerular filtration rate (eGFR) (R^2^ = 0.9519, *p* = 0.009) (Fig. 1H). Taken together, we identified ATF3 as the major responding ATF in kidney fibrosis using a non-biased approach.

**Figure 2:**
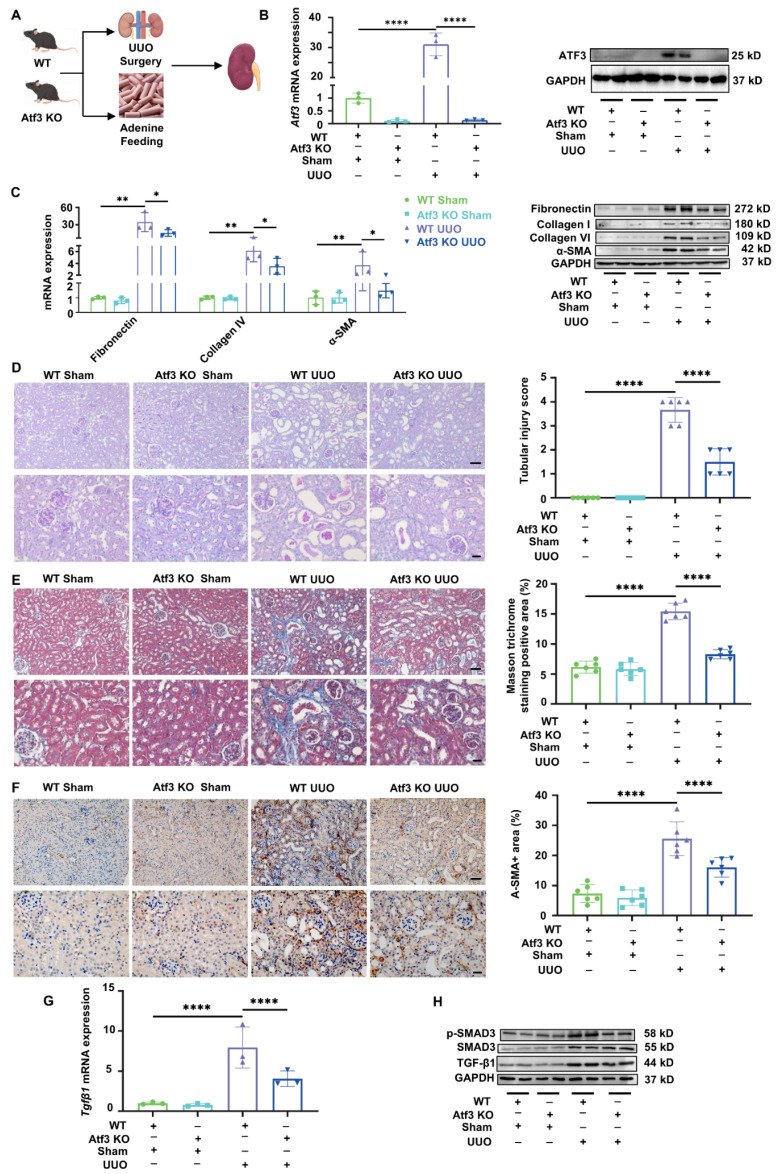
** Atf3 knockout protected against UUO-induced kidney fibrosis.** (A) Mouse modeling diagram. (B) Quantitative real-time PCR analysis and immunoblot of ATF3 in UUO kidneys. (C) Quantitative real-time PCR analysis and immunoblots of fibrotic markers in UUO kidneys. (D) Photomicrographs illustrated PAS (×200, scale bar = 50 μm / ×400, scale bar = 20 μm) staining and tubular injury scores in UUO kidneys. (E) MASSON (×200, scale bar = 50 μm /400, scale bar = 20 μm) staining with positive area measurements in UUO kidneys. (F) Immunochemistry staining (×200, scale bar = 50 μm /400, scale bar = 20 μm) of α-SMA and positive areas (%) in UUO kidneys. (G) Quantitative real-time PCR analysis of *Tgfβ1* and (H) immunoblots of TGF-β/Smad3 signaling in UUO kidneys.

TGF-β/Smad3 signaling is widely recognized as the key mediator of kidney fibrogenesis and its activation can act as a phenotype in kidney fibrosis 34-36. To determine whether ATF3 plays a functional role in kidney fibrosis, we generated ATF3 global knockout (KO) mice to perform *in vivo* based studies (Fig. 2A). In accordance with the above cross-sectional observations, we found that ATF3 protein was not expressed at the protein level in homeostatic mouse kidneys; however, it was drastically induced in the kidneys of wild-type (WT) mice following UUO surgery or adenine feeding (Fig. 2B, Supplementary Fig. S2A, S2C). Under homeostatic conditions, ATF3 knockout alone had no significant effect on kidney fibrosis (Fig. 2C-H). Knockout of ATF3 reduced the levels of profibrotic mRNAs, including *Fibronectin*, *Collagen IV* and *α-SMA* under fibrotic condition, and reduced serum creatinine and urea at the same time (Fig. 2C, Supplementary Fig. S2B). Consistently, UUO or adenine-induced elevation of fibronectin, collagen I/IV in kidneys, and α-SMA were also attenuated by ATF3 deletion (Fig. 2C, Supplementary Fig. S2D, S2G). As shown by PAS or HE staining, WT UUO/adenine mice developed severe tubular dilation, epithelial necrosis, and focal inflammatory cell infiltration in their kidneys (Fig. 2D, Supplementary Fig. S2E). ATF3 knockout markedly alleviated renal pathomorphological injury as measured in terms of tubular injury scores. As shown in Fig. 2E, the highly increased collagen positive area in tubulointerstitium was visualized using Masson staining (15.44±1.37% vs. 6.15±1.01%, *p* < 0.0001) with ATF3 deletion significantly reducing the collagen accumulation (8.29±0.77% vs. 15.44±1.37%, *p* < 0.0001) in UUO. The results in adenine-induced nephropathy were consistent with those in the UUO model (Supplementary Fig. S2F). Additionally, the upregulation of α-SMA staining by IHC was reversed by ATF3 deletion (Fig. 2F).

ATF3 inhibited increased expression of TGF-β1 and Smad3 phosphorylation (Fig. 2G-H, Supplementary Fig. S2D, Fig. S2G), thereby, repressing the TGF-β/Smad3 fibrotic signaling pathway. In summary, these results indicate that ATF3 deletion can protect against kidney fibrosis.

### ATF3 deficiency reversed *Smad7* reduction in UUO kidney, independent of direct transcriptional regulation

**Figure 3:**
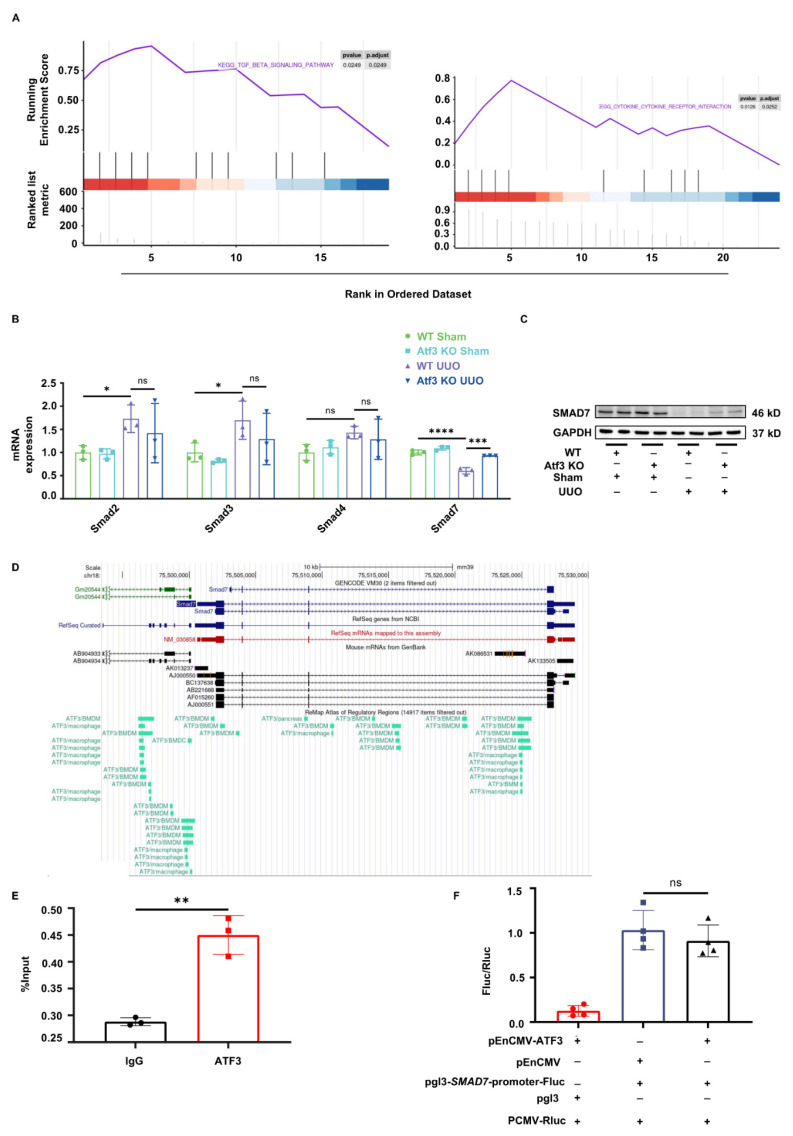
** Atf3 knockout reversed *Smad7* transcription reduction in UUO kidney by non-transcriptional manner.** (A) GSEA analysis of TGF-β signaling pathway between WT UUO and sham kidneys, between Atf3 KO UUO with WT UUO kidneys. (B) Quantitative real-time PCR analysis of *Smads* in mouse kidneys. (C) Immunoblot of SMAD7 in kidneys. (D) The UCSC genome browser database showed ATF3 was found to bind to *Smad7* gene in multiple cell lines of mouse origin. (E) ChIP-qPCR results of ATF3 to the *SMAD7* promoter in 293T cells. (F) Luciferase reporter gene assay results of ATF3 on *SMAD7* transcriptional activity. *p<0.05, **p<0.01.

We performed RNA-seq transcriptomic analysis of kidney tissues from WT and Atf3 KO mice. As shown in Supplementary Fig. S3A-B, a total of 2152 genes were significantly changed in WT UUO kidneys compared to WT sham kidneys, whereas 477 genes were significantly changed following ATF3 deletion under UUO surgery conditions. Kyoto Encyclopedia of Genes and Genomes (KEGG) pathway analysis of these differentially expressed genes revealed that the most significantly enriched pathways were primarily related to inflammation, immune regulation, and fibrosis (Supplementary Fig. S3C-D). Further gene set enrichment analysis (GSEA) suggested that TGF-β signaling pathway was upregulated in WT UUO kidneys compared to WT sham ones (*p* = 0.0249), and down-regulated in Atf3 KO UUO kidneys in comparison to WT UUO ones (*p* = 0.0126) (Fig. 3A). These results further emphasized that ATF3 deletion inhibited TGF-β/Smad3 signaling.

The TGF-β receptor complex activates the SMAD signaling pathway, including SMAD2, SMAD3, and SMAD4 to produce the full spectrum of TGF-β responses. Conversely, SMAD7 serves as a negative feedback regulator of TGF-β/Smad3 pathway 32. Considering that ATF3 is a transcription factor, we investigated whether it affected *Smad* transcription. As shown in Fig. 3B, *Smad4* mRNA levels did not differ significantly between groups. Although *Smad2* and *Smad3* were markedly induced following UUO surgery, ATF3 knockout did not significantly alter their expressions. However, *Smad7* translation level was notably reduced by UUO and was significantly reversed by ATF3 deletion (Fig. 3B). The protein levels of SMAD7 in each group were consistent with the qPCR results (Fig. 3C), indicating that ATF3 knockout can restore* Smad7* translation under fibrotic conditions.

According to the UCSC genome browser database, ATF3 binds to *Smad7* promoter regions in multiple cell lines of both human and mouse origin (Fig. 3D, Supplementary Fig. S3E). We performed ChIP-qPCR assays and found that ATF3 binds to the *SMAD7* promoter in the human kidney epithelial cell line HEK293T (Fig. 3E). Luciferase promoter reporter assay was conducted to examine the effect of ATF3 on *SMAD7* transcriptional activity (Supplementary Fig. S3F). Surprisingly, ATF3 overexpression alone had no significant effect on *SMAD7* reporter activity, indicating that ATF3 did not directly affect *SMAD7* transcription (Fig. 3F).

### ATF3 binding to HDAC6 led to reduced H3K14 acetylation in TECs under fibrotic fibrosis

Most transcription factors are thought to act by recruiting cofactors and these “coactivators” or “corepressors” are commonly involved in chromatin binding, nucleosome remodeling, and/or covalent modification of histones, thereby, regulating transcription through various mechanisms 26. Like in ATF-HDAC complex, ATFs respond to stress stimuli and recruit HDACs to specific chromatin regions for transcriptional regulation 27. Based on our previous data, we hypothesized that ATF3-HDAC interaction may reduce *Smad7* transcription.

**Figure 4:**
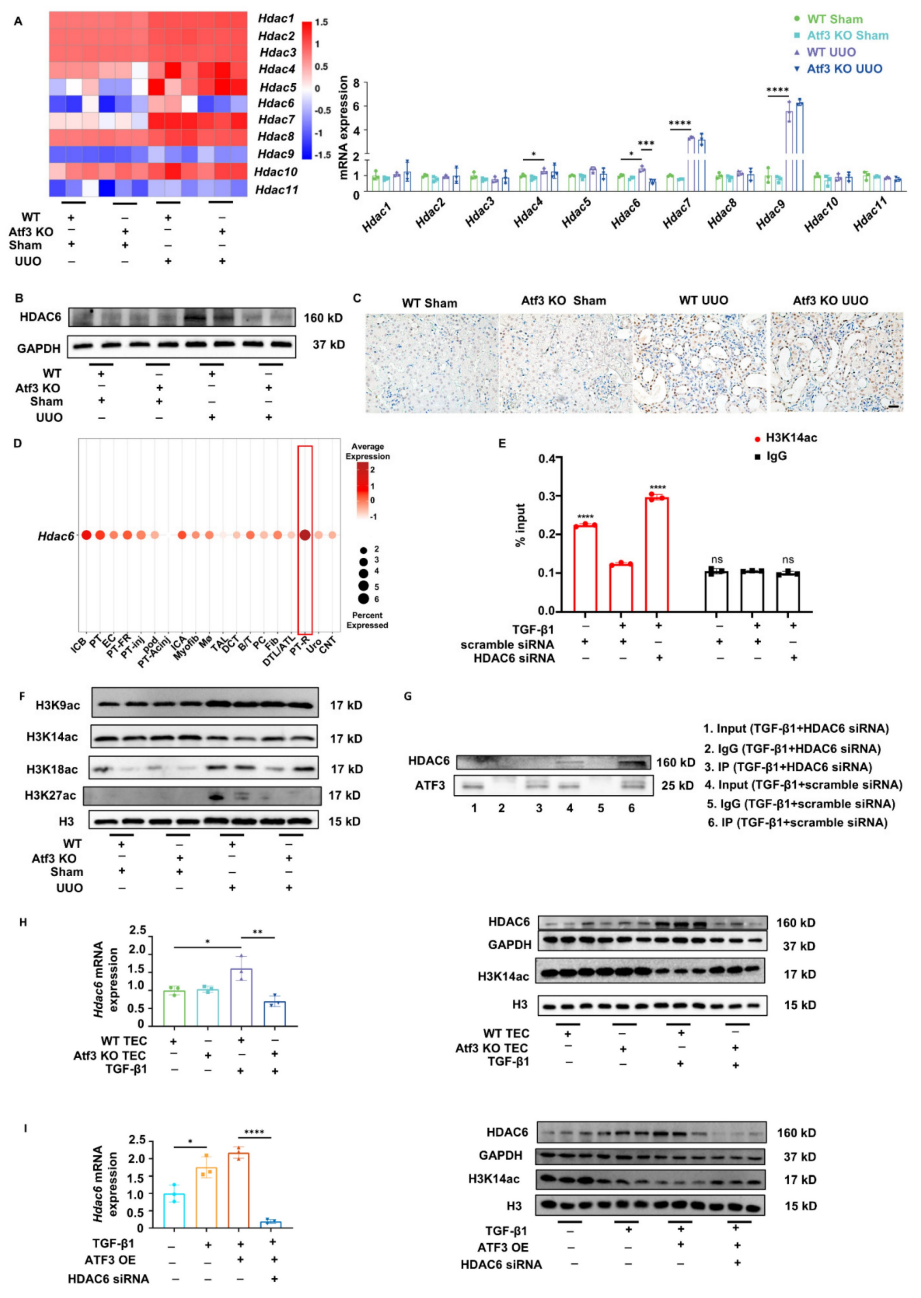
** ATF3 bound to HDAC6 in TECs under fibrotic condition, with H3K14 acetylation decreasing.** (A) Heatmap for RNA-seq and qPCR results of *Hdac 1-11* in mouse kidneys. (B) Immunoblot of HDAC6 in kidneys. (C) Immunochemistry staining (×400, scale bar = 20 μm) of HDAC6 in kidneys. (D) Single cell data showing *Hdac6* level enrichment in IRI/UUO mouse kidneys. (E) ChIP-qPCR assays results of H3K14ac to the *SMAD7* promoter in 293T cells. (F) Immunoblots of H3K9, K14, K18, K27 acetylation in kidneys. (G) The co-IP results using ATF3 antibody to bind with ATF3 or HDAC6. (H) Quantitative real-time PCR analysis of *Hdac6* and immunoblots of HDAC6/H3K14ac in primary TECs. (I) Quantitative real-time PCR analysis of *Hdac6* and immunoblots of HDAC6/H3K14ac in TCMK-1 cells. *p<0.05, **p<0.01, ***p<0.001, ****p<0.0001.

Consistent with this hypothesis, RNA-seq data and qPCR results showed that among all *Hdac*s, only *Hdac6* was elevated by UUO surgery and was reversed by ATF3 knockout (Fig. 4A). The protein levels of HDAC6 in each group were consistent with the mRNA level data and were mainly located in the TECs (Fig. 4B-C, Supplementary Fig. S4A). Single-cell data also indicated that *Hdac6* was enriched mostly in the proximal tubular epithelial cells of UUO kidneys, similar to *Atf3*. Interestingly, *Hdac6* was also induced by IRI and UUO modeling, but mostly in recovering proximal tubular epithelial cells (Fig. 4D). These results indicate that *Hdac6* specifically responds to ATF3 and might form an ATF-HDAC complex with ATF3 during kidney fibrosis. We further used TGF-β1-treated mouse kidney epithelial cell line TCMK-1 cells to mimic the proximal tubular epithelial cells under fibrotic conditions. We performed co-immunoprecipitation (co-IP) assay and observed the physical interactions between ATF3 and HDAC6 (Fig. 4G). These results confirm the formation of ATF3-HDAC6 under fibrotic conditions in proximal tubular epithelial cells.

HDACs influence transcription by inhibiting histone H3 acetylation. Literature survey indicated that HDAC6 epigenetically regulates the acetylation of histone H3 sites K9, K14, K18, and K27 37-41. Accordingly, we analyzed the acetylation levels of these sites to screen for changes in histone acetylation sites in response to fibrotic signals and ATF3 knockout. Theoretically, the ATF3 specific acetylation sites of H3 should decrease after UUO, as elevated ATF3 levels might recruit HDACs to erase the acetylation signals. As shown in Fig. 4F and Supplementary Fig. S6A, H3K9 acetylation levels did not change significantly among the groups, while H3K18 and H3K27 acetylation levels were highly increased by UUO. Only H3K14 acetylation level was significantly reduced following UUO surgery and was significantly increased by ATF3 knockout, possibly due to the deacetylation effect of HDAC6.

In addition, we observed that HDAC6 was induced by TGF-β1 in mouse primary TECs, TCMK-1, and HEK293T cell lines, with reduced H3K14 acetylation, which was reversed by ATF3 deletion or HDAC6 silence (Fig. 4H-I, Supplementary Fig. S6B-C, Fig. S7A-C). To verify that HDAC6 was responsible for changes in H3K14 acetylation levels in *Smad7* promoter, we conducted a ChIP assay. It was seen that fibrotic modeling significantly decreased H3K14ac modification within *Smad7* promoter, whereas HDAC6 deletion increased H3K14ac occupation under fibrotic conditions (Fig. 4E). Taken together, under fibrotic conditions, elevated ATF3 in TECs eliminated H3K14 acetylation in *Smad7* promoter via recruiting and binding to HDAC6.

### Elevated ATF3 in TECs aggravates kidney fibrosis via antagonizing *Smad7* transcription in an HDAC6-dependent manner

**Figure 5:**
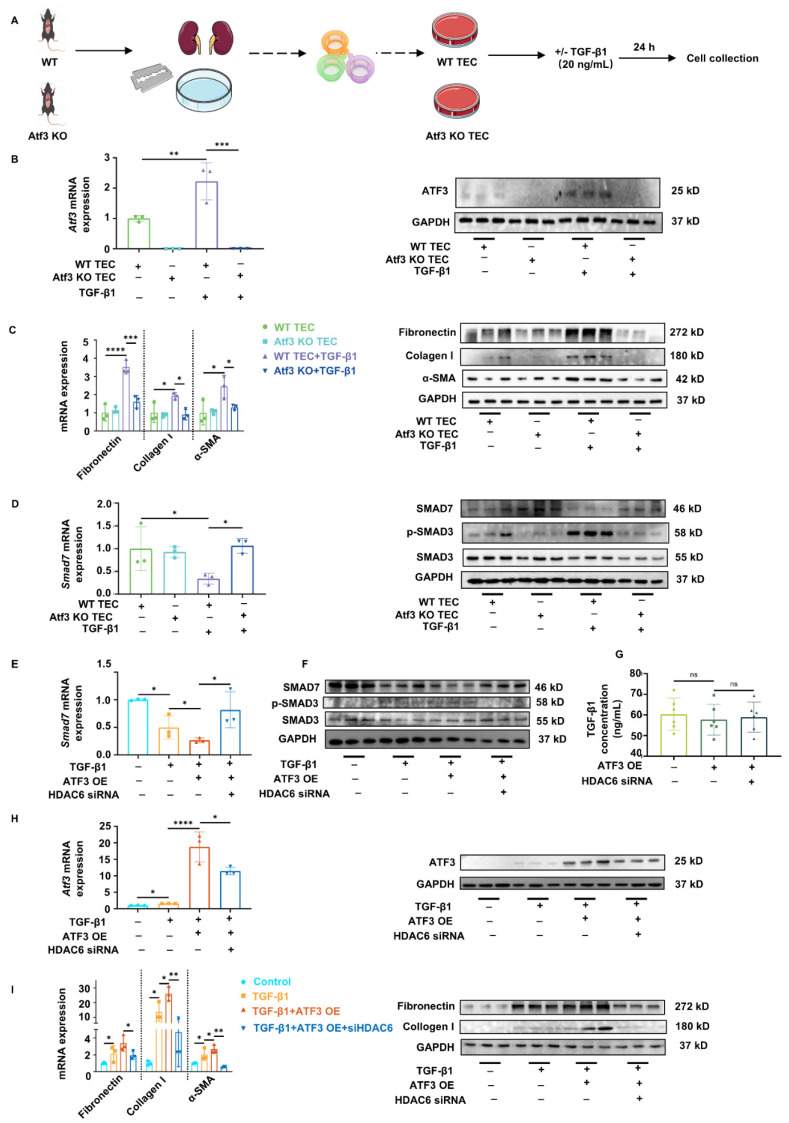
** Elevated ATF3 in TECs aggravates kidney fibrosis via antagonizing *Smad7* transcription in an HDAC6-dependent manner.** (A) The procedures and grouping of isolating primary TECs. (B) Quantitative real-time PCR analysis and immunoblot of ATF3 in primary TECs. (C) Quantitative real-time PCR analysis and immunoblots of fibrotic markers in primary TECs. (D) Quantitative real-time PCR analysis of *Smad7* and immunoblots of TGF-β/Smad3 signaling in primary TECs. (E) Quantitative real-time PCR analysis of *Smad7* in TCMK-1 cells. (F) Immunoblots of TGF-β/Smad3 signaling in TCMK-1 cells. (G) The TGF-β1 concentrations in cell culture supernatants of each TCMK-1 cell group. (H) Quantitative real-time PCR analysis and immunoblot of ATF3 in TCMK-1 cells. (I) Quantitative real-time PCR analysis and immunoblots of fibrotic markers in TCMK-1 cells. *p<0.05, **p<0.01, ***p<0.001, ****p<0.0001.

Primary TECs were isolated from WT and Atf3 KO mice. Primary TECs were then stimulated by TGF-β1 (20 ng/mL) for 24 h to mimic the *in vivo* fibrotic microenvironment (Fig. 5A). ATF3 was successfully knocked out in Atf3 KO TECs, and induced in WT TECs by TGF-β1 stimulation (Fig. 5B, Supplementary Fig. S8A). ATF3 deletion alone had no effect on the fibrotic markers. However, ATF3 deletion significantly reduced the fibrotic gene/protein levels of fibronectin, collagen I and α-SMA in TGF-β1 treated TECs (Fig. 5C, Supplementary Fig. S8A). Smad7 mRNA/protein levels were notably reduced by TGF-β1 treatment and were significantly increased by ATF3 knockout. Meanwhile, activation of TGF-β/Smad3 signaling was repressed by ATF3 knockout, indicated by TGF-β1 levels and SMAD3 phosphorylation (Fig. 5D, Supplementary Fig. S8B, left).

We then tested whether the overexpression of ATF3 could aggravate TGF-β1-induced fibrosis, and whether manipulating HDAC6 could rescue the ATF3 caused *Smad7* transcriptional changes. The TCMK-1 and HEK293T cell lines were used for transfection, stimulation, and testing. ATF3 overexpressing (OE) plasmids and HDAC6 siRNA were used to treat TCMK-1 and HEK293T cells, which were then treated with TGF-β1 (20 ng/mL) for another 24 h after transfection (Supplementary Fig. S4B). SiRNAs of mouse and human origin successfully decreased HDAC6 transcription and expression in TCMK-1 and 293T cell lines, respectively (Supplementary Fig. S5A-B). As shown in Fig. 5H and Supplementary Fig. S8C middle, gene/protein levels of ATF3 were significantly induced in TCMK-1 cells with TGF-β1 treatment and were remarkedly elevated with TGF-β1 and ATF3 OE plasmid transfection. HDAC6 silencing did not significantly affect ATF3 protein expression but reduce mRNA level. Under TGF-β1 stimulation, ATF3 overexpression exacerbated the fibrotic levels, whereas HDAC6 siRNA considerably inhibited these elevations (Fig. 5I, Supplementary Fig. S8C right). Smad7 mRNA/protein levels were significantly reduced by TGF-β1 stimulation and further reduced by ATF3 overexpression. However, they were significantly increased by HDAC6 knockdown using siRNA (Fig. 5E-F). Furthermore, TGF-β/Smad3 signaling was also activated by ATF3 overexpression and inhibited by HDAC6 siRNA (Fig. 5F, Supplementary Fig. S8B, right). The results for the same groups and treatment of 293T cells were in general consistent with those of TCMK-1 cells (Supplementary Fig. S9A-C).

We then examined whether ATF3 overexpression and HDAC6 siRNA had any effect on TGF-β1 production or any crosstalk effect on fibroblast fibrogenesis under homeostatic conditions in both TCMK-1 and 293T cell lines (Supplementary Fig. S4C). The levels of TGF-β1 in cell culture supernatants of each group did not show any significant differences (Fig. 5G). Next, the culture supernatants of TCMK-1 cells from each group were used to stimulate NRK-49F cells for 24 h to determine whether other substances could affect fibrogenesis in response to ATF3 elevation. The results showed no significant differences between the groups (Supplementary Fig. S10A-C). The results obtained with the same treatment in 293T cells were consistent with those in TCMK-1 cells (Supplementary Fig. S11 A-C, Fig. S8 C left).

### Restoration of HDAC6 targeting TECs aggravated kidney fibrosis via epigenetic control of *Smad7* expression

**Figure 6:**
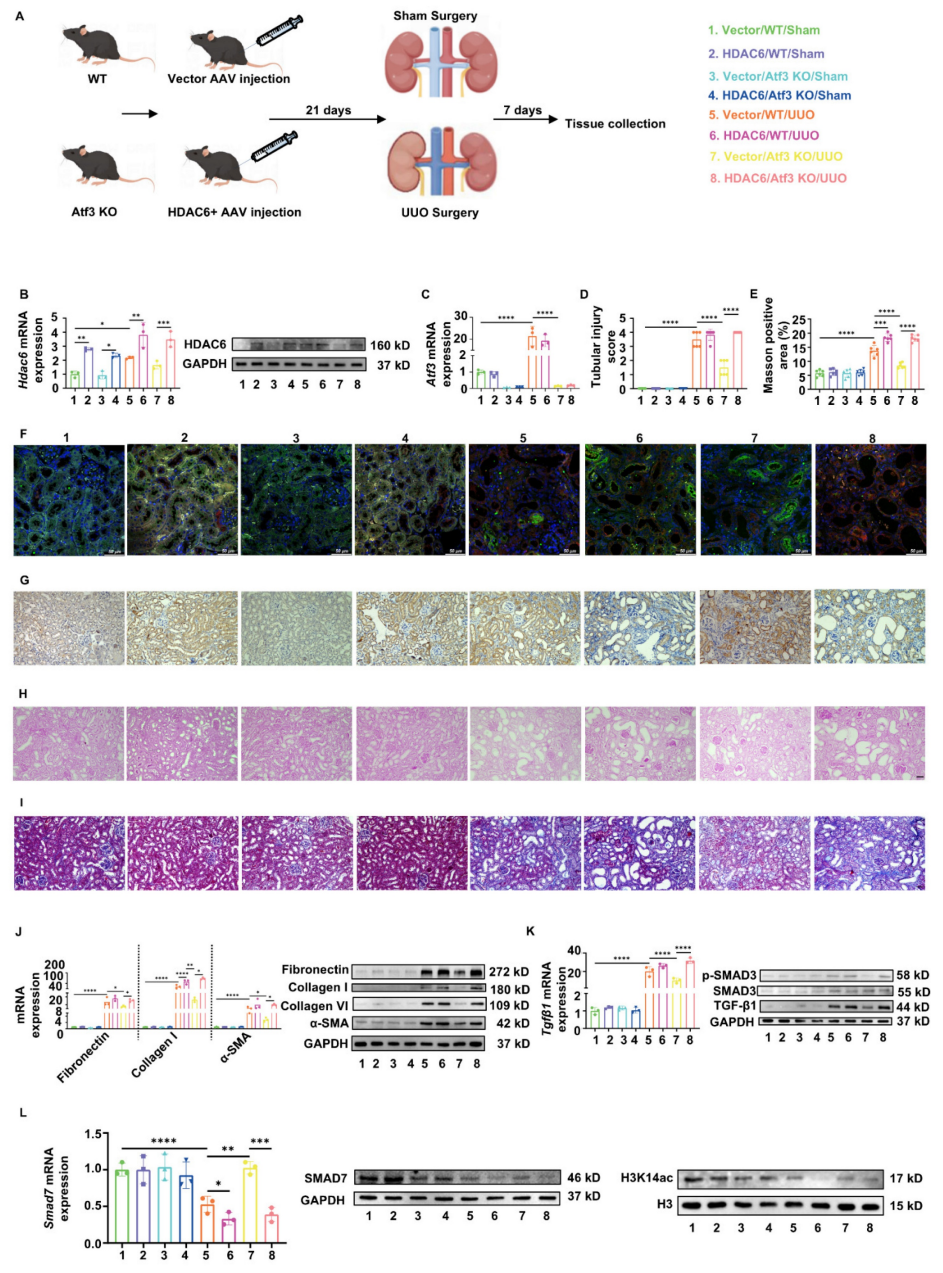
** AAV2/9-mediated restoration of HDAC6 targeting TECs aggravated kidney fibrosis.** (A) The procedures and grouping of *in vivo* restoration experiments. (B) Quantitative real-time PCR analysis and immunoblot of HDAC6 in mouse kidneys. (C) Quantitative real-time PCR analysis of *Atf3* in mouse kidneys. (F) Immunofluorescence (×400, scale bar = 50 μm) of HDAC6 in kidneys. (G) Immunochemistry staining (×200, scale bar = 50 μm) of HDAC6 in kidneys. (H) Photomicrographs illustrated PAS (×200, scale bar = 50 μm) staining and (D) tubular injury scores. (I) MASSON (×200, scale bar = 50 μm) staining with (E) positive area measurements. (J) Quantitative real-time PCR analysis and immunoblots of fibrotic markers in mouse kidneys. (K) Quantitative real-time PCR analysis of *Tgfβ1* and immunoblots of TGF-β/Smad3 signaling in mouse kidneys. (L) Quantitative real-time PCR analysis and immunoblots of SMAD7 and H3K14ac in mouse kidneys. *p<0.05, **p<0.01, ***p<0.001, ****p<0.0001.

To further elucidate the functions of the ATF3-HDAC6 complex, HDAC6 was specifically rescued in the TECs of Atf3 KO mice using AAV2/9-Ksp-cadherin-m-Hdac6-3×flag-Null (Fig. 6A). We confirmed that ectopically induced HDAC6 overexpression via AAV in mice TECs through qPCR, western blotting, immunohistochemistry and dual immunofluorescence staining with LTL (Fig. 6B, 6G, 6F), without affecting *Atf3* gene levels (Fig. 6C). Furthermore, overexpression of HDAC6 via AAV in the TECs of the sham surgery groups did not show any apparent differences (Fig. 6D-L). As shown by PAS staining (Fig. 6D, H), vector/WT/UUO mice developed tubular dilation and kidney injury, whereas HDAC6+ AAV aggravated these injuries and ATF3 knockout alleviated them. Restoration of HDAC6 expression in TECs markedly exacerbated these injuries in Atf3 KO UUO mice.

Vector/WT/UUO mice developed remarkedly increasing collagen deposition in renal tubulointerstitium as shown by Masson staining (13.40 ± 1.9% vs. 5.71 ± 1.15%, *p*<0.0001). Restoration of HDAC6 significantly aggravated the collagen accumulation (18.41 ± 1.34% vs. 13.40 ± 1.9%, *p*<0.001) while knockout of ATF3 resulted in its reduction (8.47 ± 0.94% vs. 13.40 ± 1.9%, *p*<0.0001) (Fig. 6E, I). Furthermore, HDAC6+ AAV induced increasing fibrotic marker gene/protein levels which were even higher than those induced by UUO alone. On the other hand, HDAC6 restoration in TECs also reversed fibrotic attenuation by ATF3 deletion (Fig. 6J). The TGF-β/Smad3 signaling activation in each group was consistent with the results of fibrotic markers (Fig. 6K). Meanwhile, gene and protein levels of Smad7, along with H3K14 acetylation, were reduced in vector/WT/UUO mice, and rather decreased in HDAC6/WT/UUO kidneys. Although ATF3 knockout increased these levels, HDAC6 restoration in TECs reversed them in Atf3 KO mice (Fig. 6L).

## Discussion

**Figure 7:**
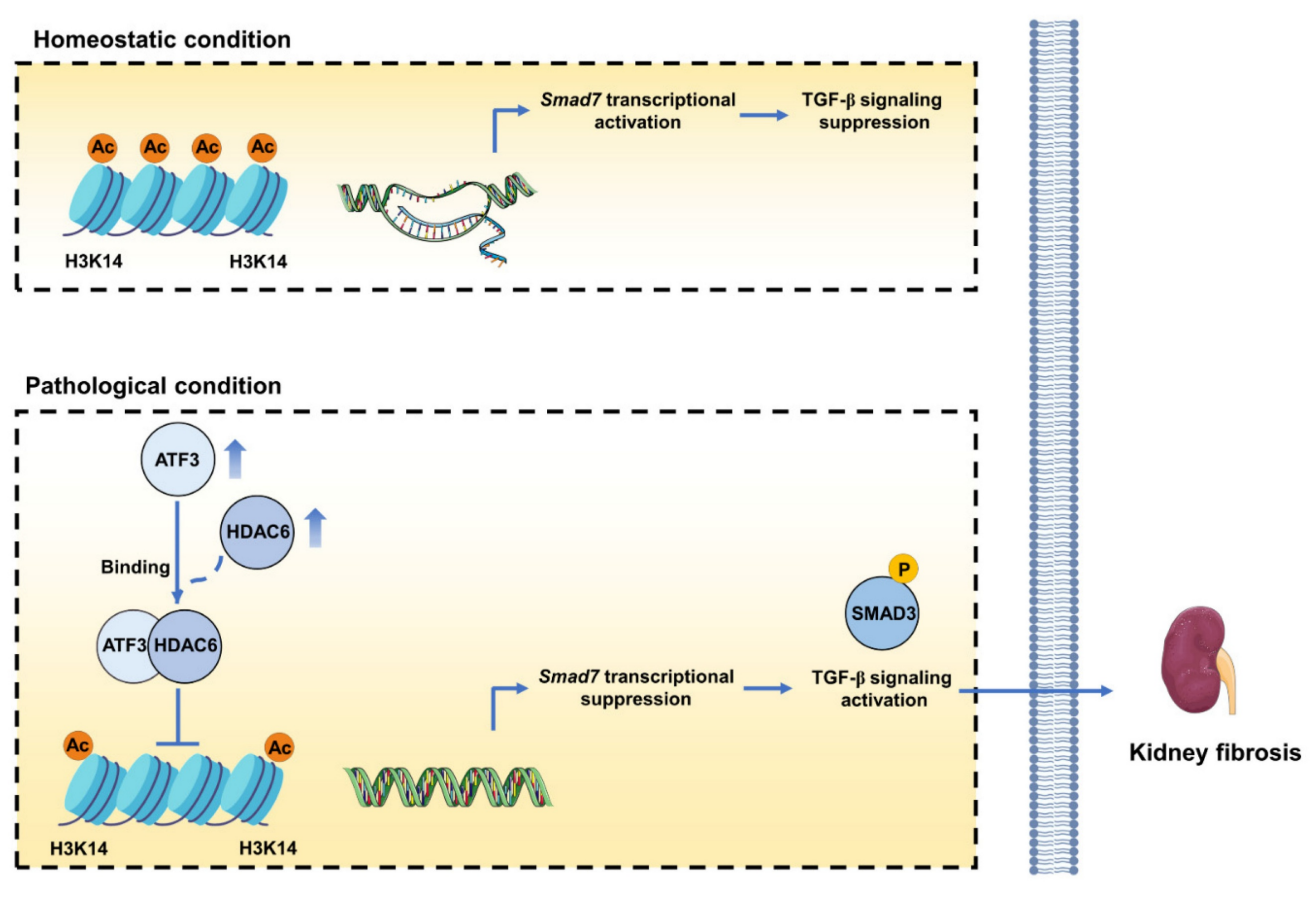
** The mechanism diagram of the study.** Upregulated ATF3 in damaged tubular epithelial cells (TECs) promotes the progression of kidney fibrosis. In TECs, under homeostatic conditions, *SMAD7* transcripts with histone 3 lysine 14 acetylation and histone opening. Under fibrotic conditions, upregulated ATF3 recruits HDAC6 to the *SMAD7* promoter, eradicating histone 3 lysine 14 acetylation (H3K14ac) and diminishing *SMAD7* transcription, thus activates TGF-β1/Smad3 signaling and aggravates kidney fibrosis.

This study identifies that ATF3 as the major ATF in kidney fibrosis, predominantly active in tubular epithelial cells (TECs). Functioning as an accomplice, ATF3 intensifies fibrotic phenotype in TECs, while its absence slows down the fibrosis process. Mechanistically, the increased ATF3 in TECs under fibrotic conditions inhibits *Smad7* transcription by recruiting HDAC6 to the *Smad7* promoter. This interaction decreases H3K14ac level and suppresses *Smad7*, thereby triggering TGF-β/Smad3 signaling (Fig. 7). Our findings elucidate ATF3's role and mechanism in kidney fibrosis, implicating its key pathogenic mechanisms for CKD.

Shi *et al* 42 reported that ATF3 is overexpressed in activated hepatic stellate cells and injured hepatocytes in fibrotic livers. ATF3 induces the expression of pro-fibrotic genes, suggesting that ATF3 can promote liver fibrosis by activating stellate cells. In addition, Li *et al* found that cardiac fibroblasts are the primary cell type expressing high ATF3 levels in response to hypertensive stimuli and that ATF3 knockout markedly exaggerated hypertensive ventricular remodeling 28. Soraya *et al* reported that transverse aortic constriction resulted in increased ATF3 expression in both cardiomyocytes and myofibroblasts, thereby, promoting a hypertrophic program and fibrotic cardiac growth, respectively 43. In contrast, we found that kidney fibrosis-induced ATF3 was mainly localized in TECs rather than in renal fibroblasts or macrophages. The single-cell data analysis also confirmed that increased ATF3 expression was mostly observed in acutely injured proximal tubular (PT) cells.

In contrast to the above-mentioned direct regulation of downstream genes, our results confirmed that kidney fibrosis-induced ATF3 in TECs recruited and directly bound with HDAC6 to the *Smad7* promoter, thus repressing *Smad7* transcription with histone H3K14 acetylation reduction. Similarly, in cardiac fibroblasts, increased ATF3 recruits and binds HDAC1 to the *Map2k3* promoter, resulting in *Map2k3* gene-associated histone deacetylation, thereby inhibiting MAP2K3 expression. The downstream signaling of MAP2K3 included inflammation and profibrotic TGF-β signaling 28. In kidneys, ATF3 induced by I/R AKI directly interacted with HDAC1 into the ATF/NF-κB sites in the *IL-6* and *IL-12b* gene promoters, and inhibited inflammation after I/R injury 17.

Following kidney injury, pro-inflammatory chemokines and danger-associated molecular patterns in proximal tubular epithelial cells can trigger an excessive inflammatory response. Uncontrolled inflammatory responses in TECs promote interstitial fibrosis 44. Additionally, maladaptive repair of injured tubules leads to kidney fibrosis 45. Liu *et al* proposed that TECs should be regarded not only as victims of kidney diseases, but also as key inflammatory and fibrogenic cells that drive kidney fibrosis 46. In response to injury, TECs can be transformed into a secretory phenotype, with the production and release of pro-inflammatory cytokines, chemokines, growth factors, and other bioactive molecules favoring inflammatory cell recruitment, fibroblast activation, and endothelial loss, which eventually drive kidney fibrosis 47. TECs induce pro-inflammatory cytokines including IL-1β, IL-6, IL-18, and so on^46^, ^47^ in response to renal injury and then cause persistent kidney injury and fibrosis development 49. Furthermore, TGF-β1 derived from injured TECs has long been considered an important pro-fibrotic growth factor 34, 51, 52. TEC is also a target of TGF-β1 as TGF-β1 could induce marked upregulation of collagen production in TECs. And autocrine TGF-β signaling increases TEC-fibroblast crosstalk 53, which induces fibroblast producing profibrotic molecules. Following injury, TECs that do not fully recover become atrophic or develop a fibrotic phenotype. This is closely associated with abnormal repair processes and fibrogenesis. Interestingly, “stress-response gene” ATF3 54 was found to be mostly located in acutely-injured TECs, while the recruited HDAC6 was mostly located in the recovering or incompletely recovered TECs, thereby playing a possible role in renal maladaptive repair and fibrogenesis. However, initial renal inflammation may be protective against AKI, while unresolved and prolonged renal inflammation may cause progressive renal fibrosis 48. When AKI occurs, inflammation and tubular epithelial repair are the first-line response to wound healing, thus the kidney has a remarkable capacity for repair 55. However, incomplete tubular repair and insistent, unresolved inflammation lead to occurrence of fibrosis 56. Inflammation in maladaptive kidney repair, whether through immune cells or renal intrinsic cells, or crosstalk among them, ultimately involves in kidney fibrosis 57. This could explain why AKI-induced ATF3 in TECs protects against AKI due to inflammation, whereas a prolonged increase in ATF3 in TECs aggravates kidney fibrosis.

Moreover, our *in vitro* studies suggested that ATF3 overexpression in TGF-β1 treated TECs upregulated fibrotic expressions, thus implying that ATF3 acted as an accomplice in injured TECs, by inhibiting *Smad7* transcription and activating TGF-β signaling. Further incubation of NRK-49F with cell culture supernatants from each group of TCMK-1/293T cell experiments without TGF-β1 treatment showed that downstream molecules of overexpressing ATF3 in homeostatic cultured TECs could not activate renal fibroblasts, which excluded possible cell crosstalk effects. HDAC6+ AAV restoration for the *in vivo* experiments and HDAC6 siRNA for the *in vitro* experiments showed that ATF3 in TECs reduced *Smad7* transcription in an HDAC6-dependent manner. Co-immunoprecipitation assays further confirmed these binding correlations. Our study may only partially explain the underlying mechanism of ATF3 in kidney fibrosis, and other possible mechanisms, including other possible cell types except for TECs, require further investigation. The mechanistic findings need future directly validation with human primary cells and larger cohorts. We used global knock of Atf3 in mice to verify and explore the mechanism, and further studies using specific knockout mice could compensate for this limitation. Additionally, the complex and dynamic changes in ATF3 expression and function during acute injury to kidney fibrosis is not investigated. The underlying mechanisms of opposite effects of ATF3 in AKI or kidney fibrosis need future exploration.

In summary, ATF3 expression was induced by kidney fibrosis and ATF3 knockout alleviated renal fibrosis. Increased ATF3 in TECs antagonized *Smad7* transcription by recruiting and binding with HDAC6 to the *Smad7* promoter region, resulting in H3K14 acetylation reduction and *Smad7* transcriptional suppression, thus activating TGF-β/Smad3 signaling. Taken together, our data shed light on the epigenetic side of renal fibrosis and provide evidence that the epigenetic interaction between ATF3 and HDAC6 might be a promising target for kidney fibrosis.

## Supplementary Material

Supplementary figures and tables.
