# Supplementary material for: ATF3 aggravates kidney fibrosis via HDAC6-dependent epigenetic reprogramming
Source: Int J Biol Sci. 2026 Jan 1;22(2):841–57. doi: 10.7150/ijbs.125062 (PMC12781174; doi:10.7150/ijbs.125062)
Supplement: Supplementary file 1 — Supplementary figures and tables. [file ijbsv22p0841s1.pdf]

Table S1. Antibodies used in the study.

| Antibody name&species | Company | Cargo number |
|---|---|---|
| Rabbit Anti-ATF3 | Cell Signaling Technology | 33593 |
| Rabbit Anti-HDAC6 | Cell Signaling Technology | 7612S |
| Rabbit Anti-H3 | Huabio Technology | ET1701-64 |
| Rabbit Anti-H3K9ac | Cell Signaling Technology | 9649T |
| Rabbit Anti-H3K14ac | Huabio Technology/ Active motif | ET1706/3959 |
| Rabbit Anti-H3K18ac | Huabio Technology | HA500047 |
| Rabbit Anti-H3K27ac | Cell Signaling Technology | 8173S |
| Rabbit Anti-α-SMA | Abcam | ab5694 |
| Rabbit Anti-Collagen I | Boster Biological Technology | PB0981 |
| RabbitAnti-Fibronectin | Affinity Biosciences | AF5335 |
| RabbitAnti-Collagen VI | Huabio Technology | ET1612-91 |
| Mouse Anti-TGF-β1 | Affinity Biosciences | BF8012 |
| Rabbit Anti- phospho-Smad3 | Huabio Technology | ET1607-43 |
| Rabbit Anti-Smad3 | Huabio Technology | ET1609-41 |
| Rabbit Anti-Smad7 | Huabio Technology | ER1912-61 |
| Rabbit Anti-GAPDH | Affinity Biosciences | AF7021 |

Table S2. Primer sequences used in qPCR.

| Gene | Sequences |
|---|---|
| *Hprt* | Forward5'-TCAGTCAACGGGGGACATAAA-3'<br>Reverse5'-GGGGCTGTACTGCTTAACCAG-3' |
| *Atf3* | Forward5'-TTTGCTAACCTGACACCCTTTG-3'<br>Reverse5'-AGAGGACATCCGATGGCAGA-3' |
| *Tgfb1* | Forward5'-CCACCTGCAAGACCATCGAC -3'<br>Reverse5'-CTGGCGAGCCTTAGTTTGGAC -3' |
| *α-sma* | Forward5'-CCCAGACATCAGGGAGTAATGG -3'<br>Reverse5'-TCTATCGGATACTTCAGCGTCA-3' |
| *Collagen I* | Forward5'-GCTCCTCTTAGGGGCCACT-3'<br>Reverse5'-ATTGGGGACCCTTAGGCCAT-3' |
| *Collagen IV* | Forward5'-CCTGGCACAAAAGGGACGA-3'<br>Reverse5'-ACGTGGCCGAGAATTTCACC-3' |
| *Fibronectin* | Forward 5'-ATGTGGACCCCTCCTGATAGT-3'<br>Reverse 5'-GCCCAGTGATTTCAGCAAAGG-3' |
| *Smad2* | Forward 5'-AAGCCATCACCACTCAGAATTG-3'<br>Reverse 5'-CACTGATCTACCGTATTTGCTGT-3' |
| *Smad3* | Forward 5'-AGGGGCTCCCTCACGTTATC-3'<br>Reverse 5'-CATGGCCCGTAATTCATGGTG-3' |
| *Smad4* | Forward 5'-ACACCAACAAGTAACGATGCC-3'<br>Reverse 5'-GCAAAGGTTTCACTTTCCCCA-3' |
| *Smad7* | Forward 5'-TTTTCTCAAACCAACTGCAGGC-3'<br>Reverse 5'-AATTGAGCTGTCCGAGGCAA-3' |
| *Hdac1* | Forward 5'-TGAAGCCTCACCGAATCCG-3'<br>Reverse 5'-GGGCGAATAGAACGCAGGA-3' |
| *Hdac2* | Forward 5'-GGAGGAGGCTACACAATCCG-3'<br>Reverse 5'-TCTGGAGTGTTCTGGTTTGTCA-3' |
| *Hdac3* | Forward 5'- GCCAAGACCGTGGCGTATT-3'<br>Reverse 5'-GTCCAGCTCCATAGTGGAAGT-3' |

| | |
|---|---|
| *Hdac4* | Forward 5’-CTGCAAGTGGCCCCTACAG-3’<br>Reverse 5’-CTGCTCATGTTGACGCTGGA-3’ |
| *Hdac5* | Forward 5’-AGCACCGAGGTAAAGCTGAG-3’<br>Reverse 5’-GAACTCTGGTCCAAAGAAGCG-3’ |
| *Hdac6* | Forward 5’-TCCACCGGCCAAGATTCTTC-3’<br>Reverse 5’-GCCTTTCTTCTTTACCTCCGCT-3’ |
| *Hdac7* | Forward 5’-TTCCTGGCAGGCTTACACC-3’<br>Reverse 5’-ATGGACTGTTCTCTCAAGGGC-3’ |
| *Hdac8* | Forward 5’-ACTATTGCCGGAGATCCAATGT-3’<br>Reverse 5’-CCTCCTAAAATCAGAGTTGCCAG-3’ |
| *Hdac9* | Forward 5’-GCGGTCCAGGTTAAAACAGAA-3’<br>Reverse 5’-GCCACCTCAAACACTCGCTT-3’ |
| *Hdac10* | Forward 5’-ACAGCCACTCGACTGCTCT-3’<br>Reverse 5’-GATGCCTCACAAGCTGACAAA-3’ |
| *Hdac11* | Forward 5’-GTGTACTCACCACGTTACAACA-3’<br>Reverse 5’-GCTCGTTGAGATAGCGCCTC-3’ |

Table S3. Clinical data of patients with different causes of CKD.

| Patient | Age at biopsy (yr) | Gender | Diagnoses | Scr (μmol/L) | eGFR (mL/min/1.73m$^2$) |
|---|---|---|---|---|---|
| 1 | 48 | M | RCC | 78 | 97.86 |
| 2 | 44 | M | MN | 95 | 83.53 |
| 3 | 30 | F | IgAN | 91 | 73.17 |
| 4 | 28 | F | FSGS | 153 | 39.54 |
| 5 | 50 | F | AAV | 344 | 12.74 |
| 6 | 48 | F | DN | 82 | 73.65 |

Scr, serum creatinine; eGFR, estimated glomerular filtration rate; F, female; M, male; RCC, renal cell carcinoma; MN, membranous nephropathy; IgAN, IgA nephropathy; FSGS, focal segmental glomerulosclerosis; AAV, anti-neutrophil cytoplasmic antibody-associated vasculitis; DN, diabetic nephropathy.

Table S4. Primer sequences used in ChIP-qPCR.

| Gene | Sequences |
|---|---|
| *SMAD7* | Forward5'-CCGCCAGCTTGGGTATATGT-3'<br>Reverse5'-GTTTCTGGGCCTCGTAGCTT-3' |

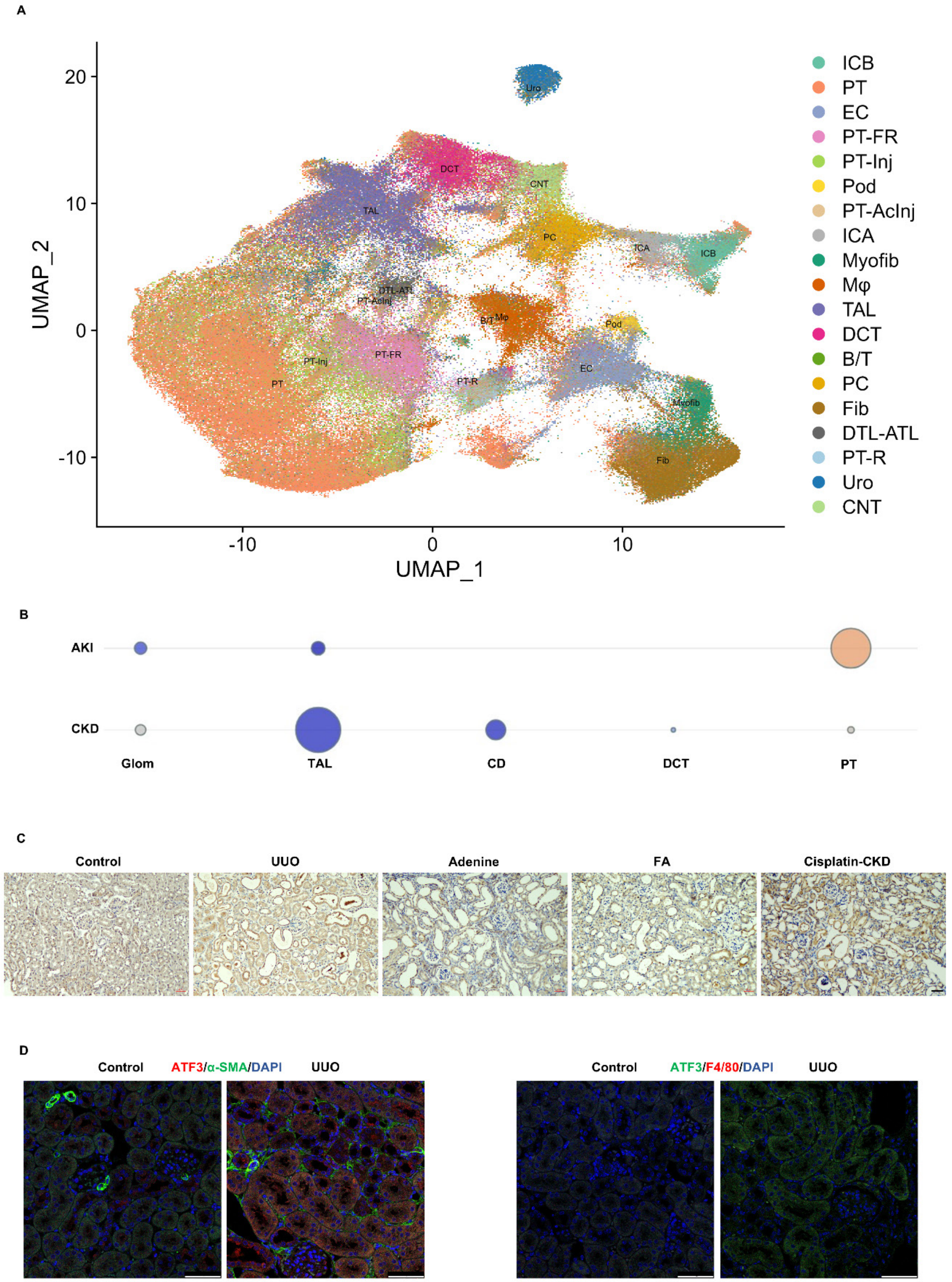


**Figure S1. ATF3 was the major responding ATF in renal interstitial fibrosis and was**

**induced in tubular epithelial cells.** (A) UMAP for single cell data in IRI/UUO mouse kidneys. (B) Kidney tissue atlas from Kidney Precision Medicine Project showing ATF3 enrichment in CKD patients' kidneys. (C) Immunochemistry staining (×200, scale bar = 50 μm) of ATF3 in UUO, adenine, FA and cisplatin-CKD mouse kidneys and relative controls. (D) Immunofluorescence (×400, scale bar = 50 μm) of ATF3 double-stained with in UUO and control kidneys.

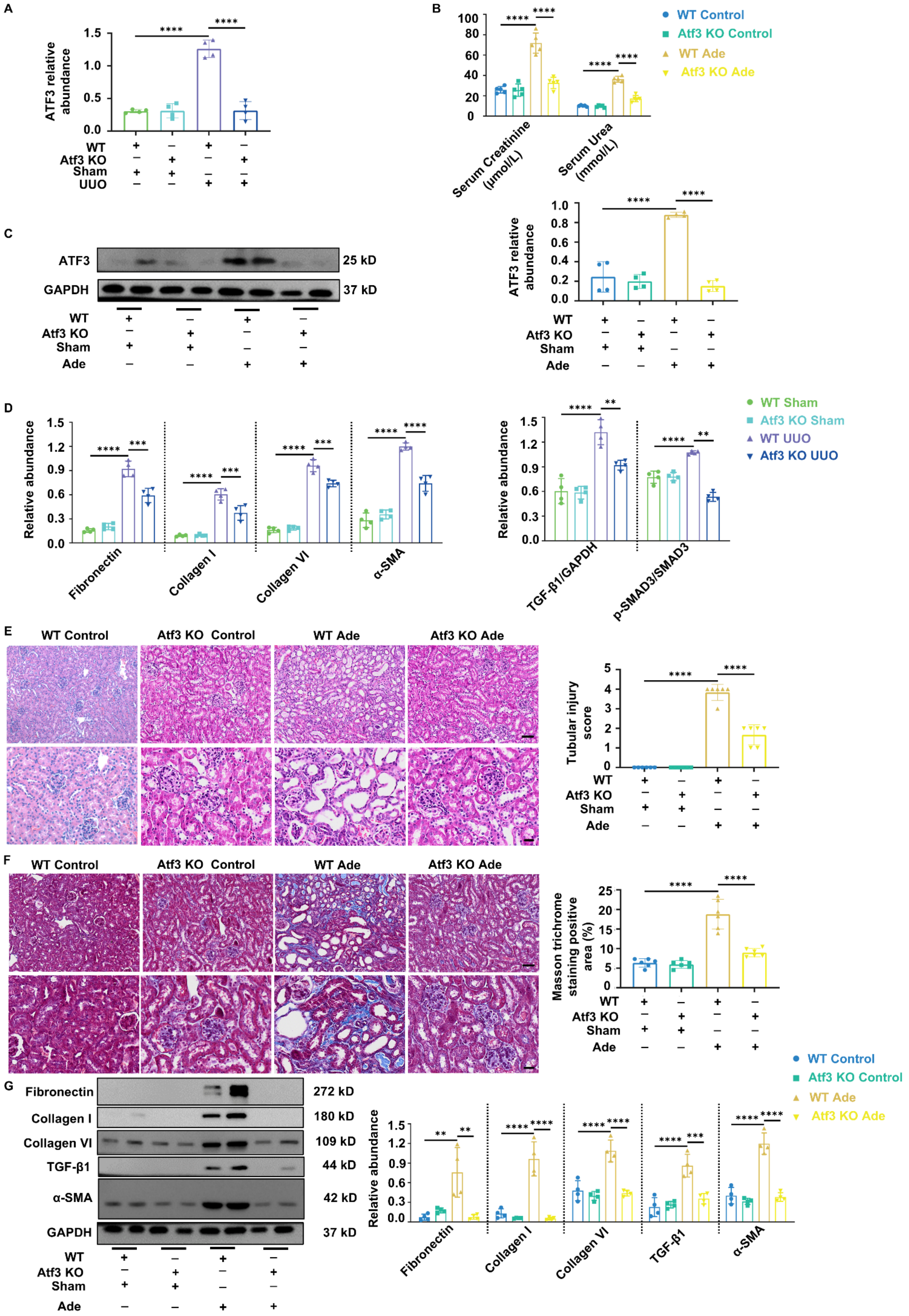
A
ATF3 relative abundance
1.5
1.0
0.5
0.0
****
****
WT
Atf3 KO
Sham
UUO
B
WT Control
Atf3 KO Control
WT Ade
Atf3 KO Ade
100
80
60
40
20
0
Serum Creatinine (μmol/L)
Serum Urea (mmol/L)
C
ATF3
GAPDH
25 kD
37 kD
WT
Atf3 KO
Sham
Ade
ATF3 relative abundance
1.0
0.8
0.6
0.4
0.2
0.0
D
Relative abundance
1.5
1.2
0.9
0.6
0.3
0.0
Fibronectin
Collagen I
Collagen VI
α-SMA
TGF-β1/GAPDH
p-SMAD3/SMAD3
WT Sham
Atf3 KO Sham
WT UUO
Atf3 KO UUO
E
WT Control
Atf3 KO Control
WT Ade
Atf3 KO Ade
Tubular injury score
5
4
3
2
1
0
F
WT Control
Atf3 KO Control
WT Ade
Atf3 KO Ade
Masson trichrome staining positive area (%)
25
20
15
10
5
0
G
Fibronectin
Collagen I
Collagen VI
TGF-β1
α-SMA
GAPDH
272 kD
180 kD
109 kD
44 kD
42 kD
37 kD
WT
Atf3 KO
Sham
Ade
WT Control
Atf3 KO Control
WT Ade
Atf3 KO Ade
Relative abundance
1.5
1.2
0.9
0.6
0.3
0.0
Fibronectin
Collagen I
Collagen VI
TGF-β1
α-SMA

**Figure S2. Atf3 knockout protected against UUO/adenine-induced renal interstitial fibrosis and suppressed TGF-β/Smad3 signaling.** (A) Immunoblot scoring of ATF3 in UUO kidneys. (B) Serum creatinine and urea in adenine nephropathy. (C) Immunoblot and scoring of ATF3 in adenine kidneys. (D) Immunoblot scorings of fibrotic markers and TGF-β/Smad3 signaling in UUO kidneys. (E) Photomicrographs illustrated HE (×200, scale bar = 50 μm /400, scale bar = 20 μm) staining and tubular injury scores in adenine kidneys. (F) MASSON (×200, scale bar = 50 μm /400, scale bar = 20 μm) staining with positive area measurements in adenine kidneys. (G) Immunoblots and scorings of fibrotic markers and TGF-β/Smad3 signaling in adenine kidneys.

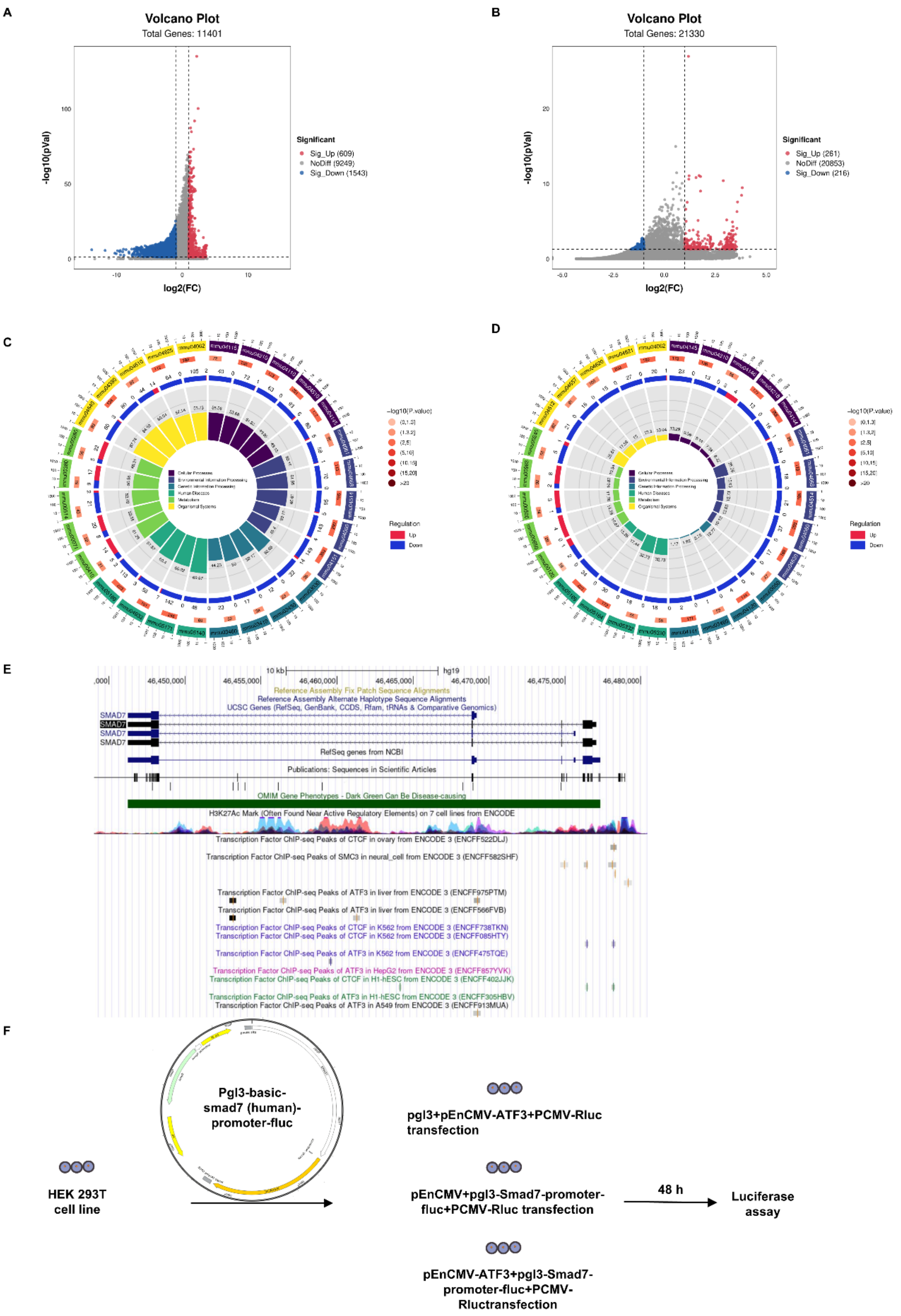

A
Volcano Plot
Total Genes: 11401
-log10(pVal)
log2(FC)
Significant
Sig_Up (609)
NoDiff (9249)
Sig_Down (1543)
B
Volcano Plot
Total Genes: 21330
-log10(pVal)
log2(FC)
Significant
Sig_Up (261)
NoDiff (20853)
Sig_Down (216)
C
D
-log10(P.value)
Regulation
Up
Down
E
10 kb
hg19
46,450,000
46,455,000
46,460,000
46,465,000
46,470,000
46,475,000
46,480,000
Reference Assembly Fix Patch Sequence Alignments
Reference Assembly Alternate Haplotype Sequence Alignments
UCSC Genes (RefSeq, GenBank, CCDS, Rfam, tRNAs & Comparative Genomics)
SMAD7
SMAD7
SMAD7
SMAD7
RefSeq genes from NCBI
Publications: Sequences in Scientific Articles
OMIM Gene Phenotypes - Dark Green Can Be Disease-causing
H3K27Ac Mark (Often Found Near Active Regulatory Elements) on 7 cell lines from ENCODE
Transcription Factor ChIP-seq Peaks of CTCF in ovary from ENCODE 3 (ENCFF522DLJ)
Transcription Factor ChIP-seq Peaks of SMC3 in neural_cell from ENCODE 3 (ENCFF582SHF)
Transcription Factor ChIP-seq Peaks of ATF3 in liver from ENCODE 3 (ENCFF975PTM)
Transcription Factor ChIP-seq Peaks of ATF3 in liver from ENCODE 3 (ENCFF566FVB)
Transcription Factor ChIP-seq Peaks of CTCF in K562 from ENCODE 3 (ENCFF738TKN)
Transcription Factor ChIP-seq Peaks of CTCF in K562 from ENCODE 3 (ENCFF085HTY)
Transcription Factor ChIP-seq Peaks of ATF3 in K562 from ENCODE 3 (ENCFF475TQE)
Transcription Factor ChIP-seq Peaks of ATF3 in HepG2 from ENCODE 3 (ENCFF857YVK)
Transcription Factor ChIP-seq Peaks of CTCF in H1-hESC from ENCODE 3 (ENCFF402JJK)
Transcription Factor ChIP-seq Peaks of ATF3 in H1-hESC from ENCODE 3 (ENCFF305HBV)
Transcription Factor ChIP-seq Peaks of ATF3 in A549 from ENCODE 3 (ENCFF913MUA)
F
Pgl3-basic-smad7 (human)-promoter-fluc
HEK 293T cell line
pgl3+pEnCMV-ATF3+PCMV-Rluc transfection
pEnCMV+pgl3-Smad7-promoter-fluc+PCMV-Rluc transfection
pEnCMV-ATF3+pgl3-Smad7-promoter-fluc+PCMV-Rluctransfection
48 h
Luciferase assay

**Figure S3. Volcano plots, KEGG analysis, and ATF3 binding data.** (A) Volcano plots between WT sham vs. UUO kidneys. (B) Volcano plots between WT UUO vs. Atf3 KO UUO kidneys. (C) KEGG analysis between WT sham vs. UUO kidneys. (D) KEGG analysis between WT UUO vs. Atf3 KO UUO kidneys. (E) The UCSC genome browser database showed ATF3 was found to bind to *Smad7* gene in multiple cell lines of human origin. (F) Luciferase reporter gene assay procedure of ATF3 on *SMAD7* transcriptional activity.

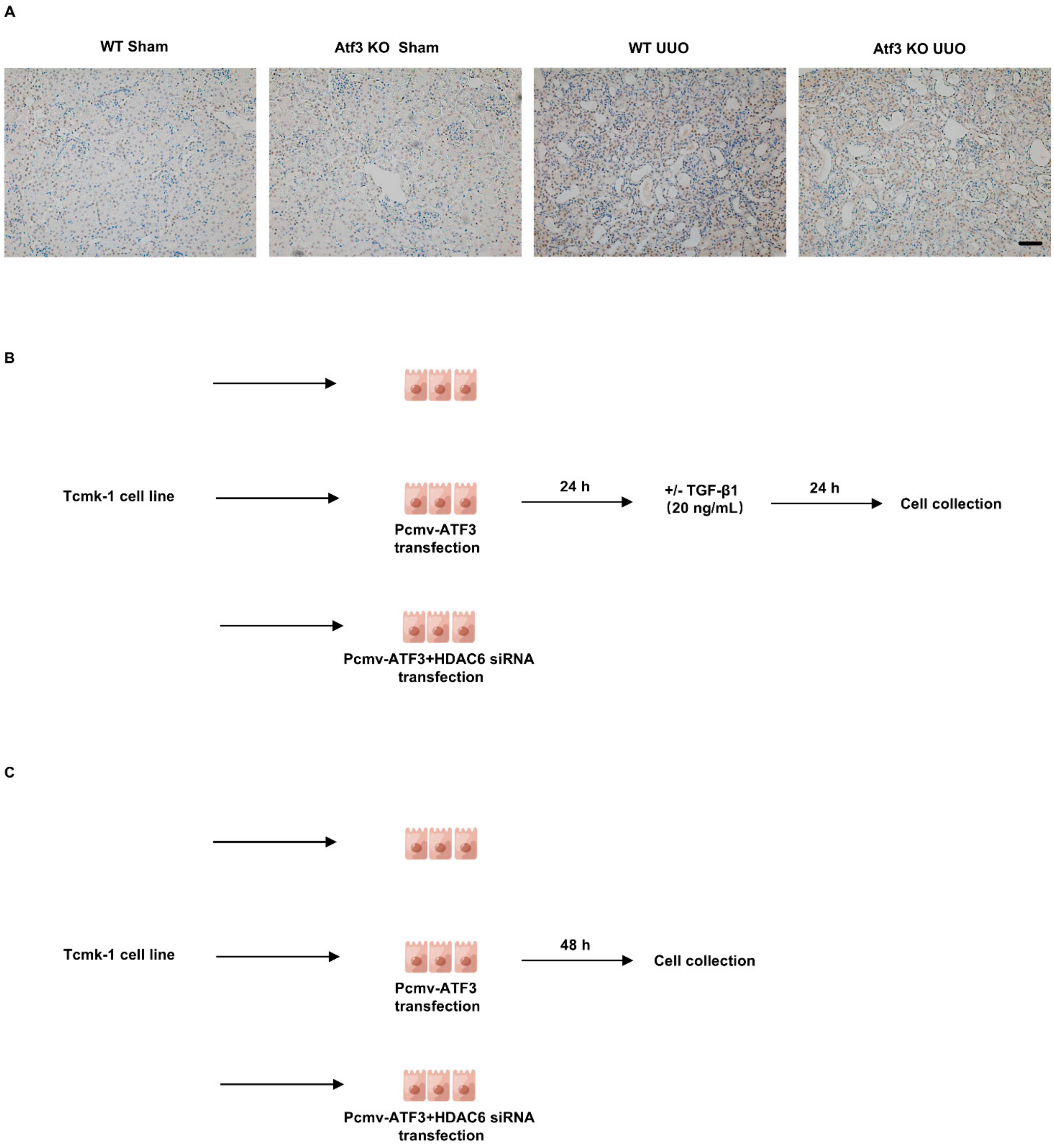
A
WT Sham
Atf3 KO Sham
WT UUO
Atf3 KO UUO
B
Tcmk-1 cell line
Pcmv-ATF3
transfection
24 h
+/- TGF-β1
(20 ng/mL)
24 h
Cell collection
Pcmv-ATF3+HDAC6 siRNA
transfection
C
Tcmk-1 cell line
Pcmv-ATF3
transfection
48 h
Cell collection
Pcmv-ATF3+HDAC6 siRNA
transfection

**Figure S4. IHC staining of HDAC6 in kidneys and TCMK-1 cell grouping.** (A) Immunochemistry staining (×200, scale bar = 50 μm) of HDAC6 in kidneys. (B) The procedures and grouping of TCMK-1 cells with TGF-β1 stimulation. (C)The procedures and grouping of TCMK-1 cells without TGF-β1 stimulation.

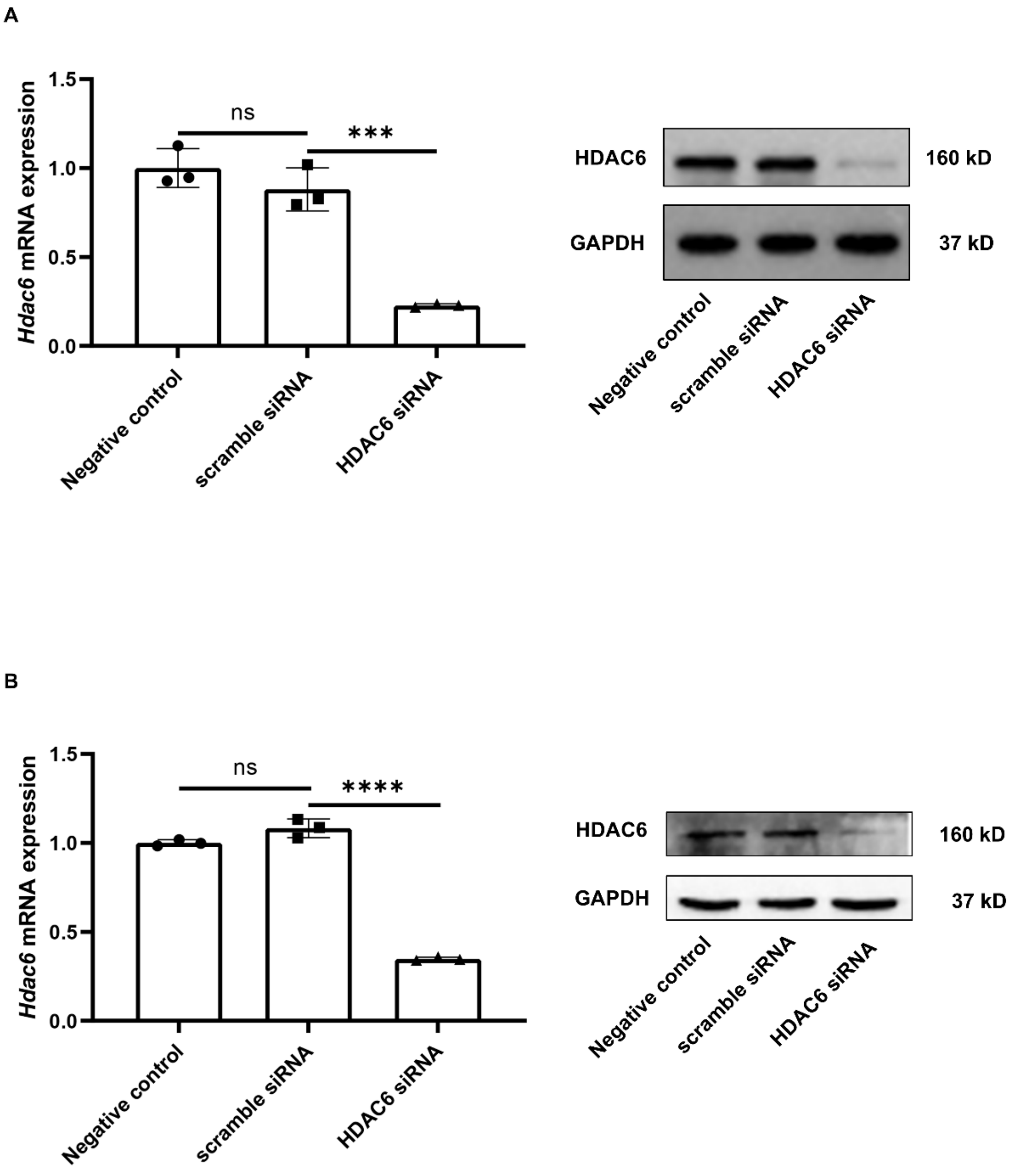


**Figure S5. The siRNAs from mouse and human origin both successfully decreased**

**HDAC6 transcription and expression in TCMK-1 and 293T cell line, respectively.** (A) Quantitative real-time PCR analysis and immunoblot of HDAC6 in TCMK-1 cells. (B) Quantitative real-time PCR analysis and immunoblot of HDAC6 in 293T cells. ***$p<0.001$, ****$p<0.0001$.

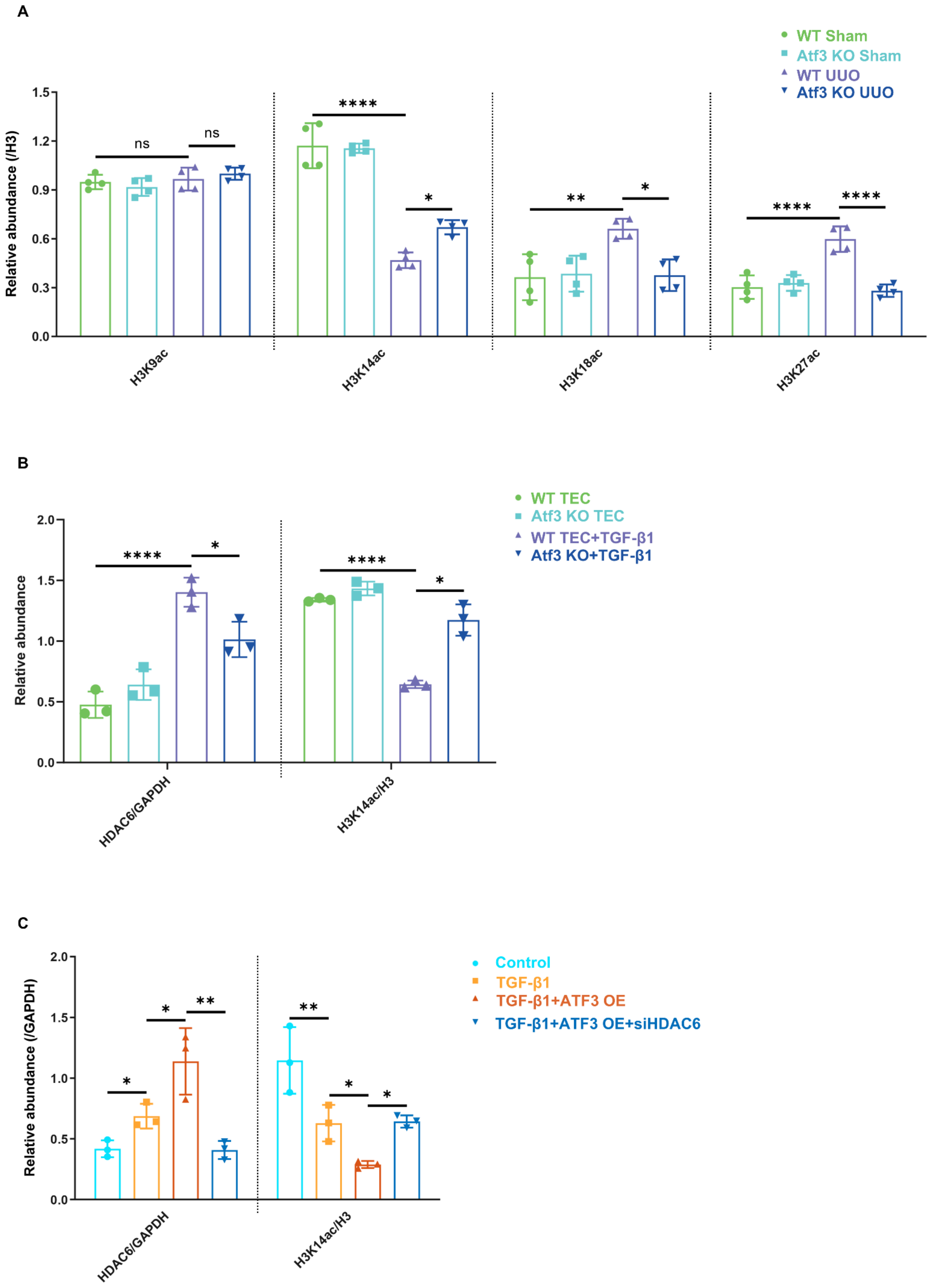
A
WT Sham
Atf3 KO Sham
WT UUO
Atf3 KO UUO
Relative abundance (/H3)
1.5
1.2
0.9
0.6
0.3
0.0
ns
ns
****
*
**
*
****
****
H3K9ac
H3K14ac
H3K18ac
H3K27ac
B
WT TEC
Atf3 KO TEC
WT TEC+TGF-β1
Atf3 KO+TGF-β1
Relative abundance
2.0
1.5
1.0
0.5
0.0
****
*
****
*
HDAC6/GAPDH
H3K14ac/H3
C
Control
TGF-β1
TGF-β1+ATF3 OE
TGF-β1+ATF3 OE+siHDAC6
Relative abundance (/GAPDH)
2.0
1.5
1.0
0.5
0.0
*
*
**
*
**
*
*
HDAC6/GAPDH
H3K14ac/H3

**Figure S6. Immunoblot scorings of multiple acetylation locations in H3 in UUO kidneys, primary TECs or TCMK-1 cells.** (A) Immunoblot scorings of H3K9ac, H3K14ac, H3K18ac, H3K27ac in UUO kidneys. (B) Immunoblot scorings of HDAC6, H3K14ac in primary TECs. (C) Immunoblot scorings of HDAC6, H3K14ac in TCMK-1 cells. *p<0.05, **p<0.01, ****p<0.0001.

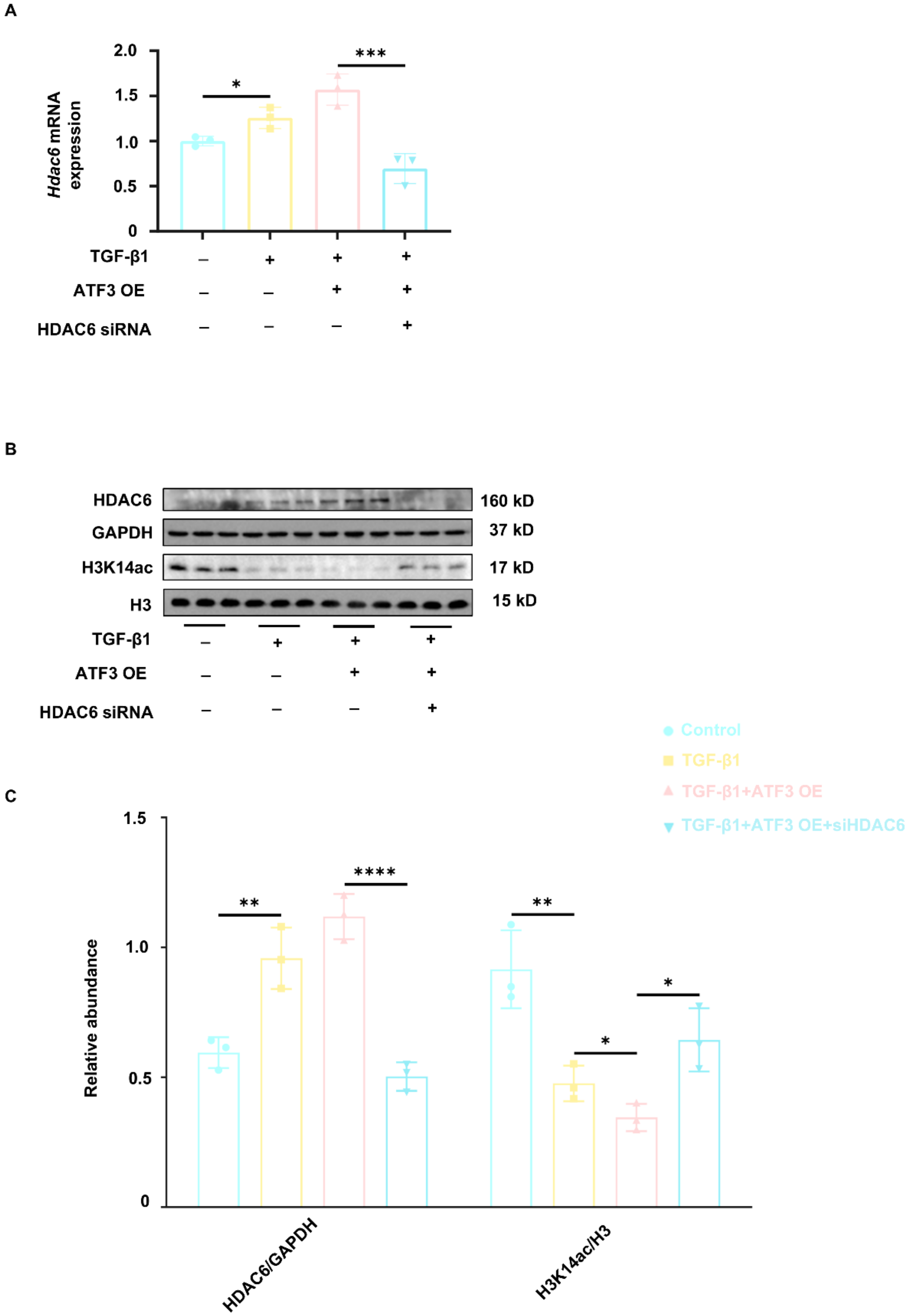
A
2.0
1.5
1.0
0.5
0
Hdac6 mRNA expression
*
***
TGF-β1
ATF3 OE
HDAC6 siRNA
B
HDAC6
GAPDH
H3K14ac
H3
160 kD
37 kD
17 kD
15 kD
TGF-β1
ATF3 OE
HDAC6 siRNA
C
Control
TGF-β1
TGF-β1+ATF3 OE
TGF-β1+ATF3 OE+siHDAC6
1.5
1.0
0.5
0
Relative abundance
**
****
**
*
*
HDAC6/GAPDH
H3K14ac/H3

**Figure S7. Quantitative real-time PCR analysis of *HDAC6* and immunoblots of HDAC6/H3K14ac in 293T cells.** *p<0.05, **p<0.01, ***p<0.001, ****p<0.0001.

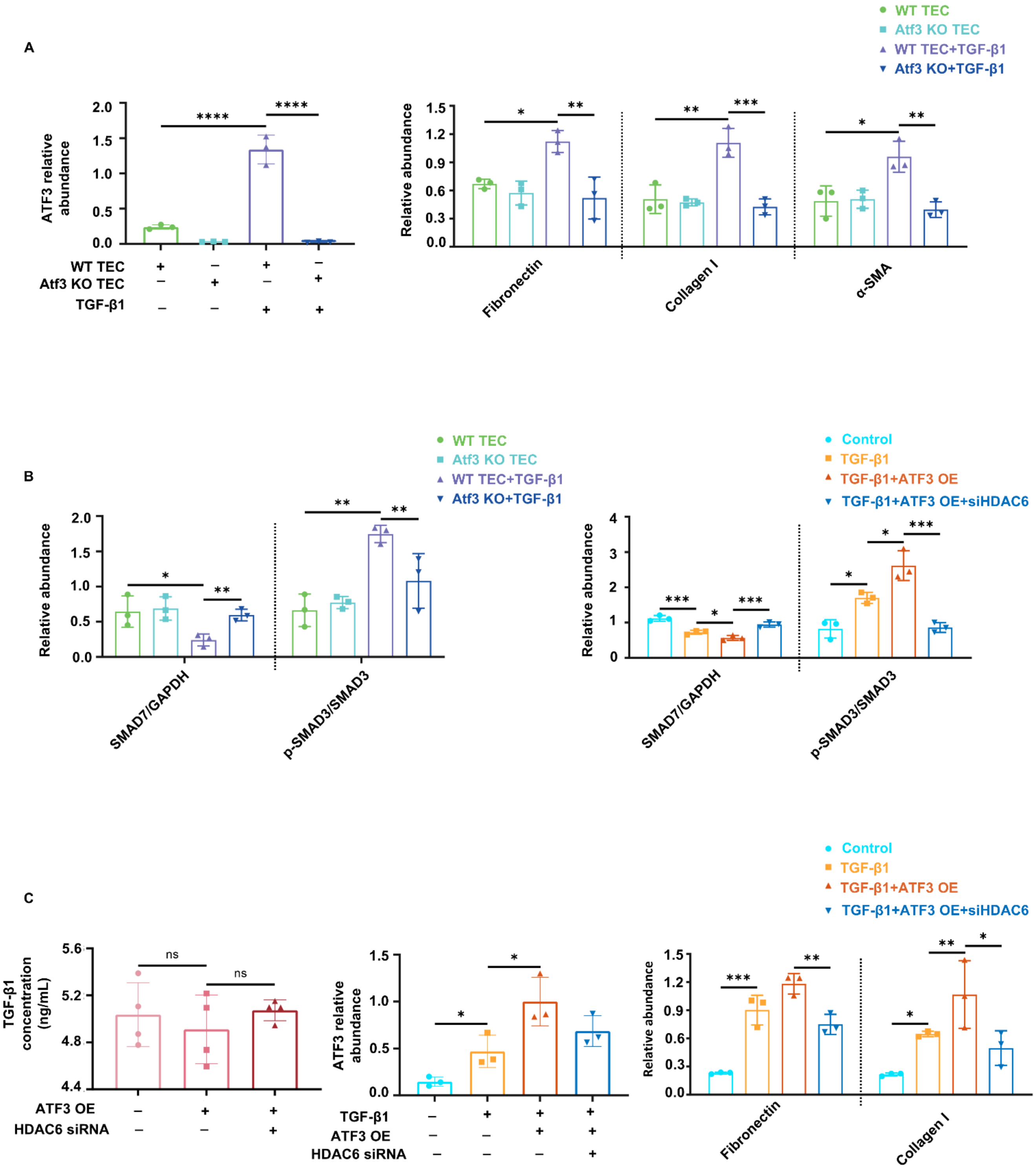
A
WT TEC
Atf3 KO TEC
WT TEC+TGF-β1
Atf3 KO+TGF-β1
ATF3 relative abundance
2.0
1.5
1.0
0.5
0.0
****
****
WT TEC + – + –
Atf3 KO TEC – + – +
TGF-β1 – – + +
Relative abundance
1.5
1.2
0.9
0.6
0.3
0.0
*
**
***
Fibronectin
Collagen I
α-SMA
B
WT TEC
Atf3 KO TEC
WT TEC+TGF-β1
Atf3 KO+TGF-β1
Relative abundance
SMAD7/GAPDH
p-SMAD3/SMAD3
Control
TGF-β1
TGF-β1+ATF3 OE
TGF-β1+ATF3 OE+siHDAC6
C
TGF-β1 concentration (ng/mL)
5.6
5.2
4.8
4.4
ns
ATF3 OE – + +
HDAC6 siRNA – – +
ATF3 relative abundance
TGF-β1 – + + +
ATF3 OE – – + +
HDAC6 siRNA – – – +
Fibronectin
Collagen I

**Figure S8. Immunoblot scorings in primary TECs / TCMK-1 cells and the TGF-β1 concentrations in supernatants of each 293T cell group.** (A) Immunoblot scoring of ATF3 and fibrotic markers in primary TECs. (B) Immunoblot scoring of TGF-β/Smad3 signaling in primary TECs and TCMK-1 cells. (C) The TGF-β1 concentrations in supernatants of each 293T cell group. Immunoblot scoring of ATF3 and fibrotic markers in TCMK-1 cells. *$p<0.05$, **$p<0.01$, ***$p<0.001$, ****$p<0.0001$.

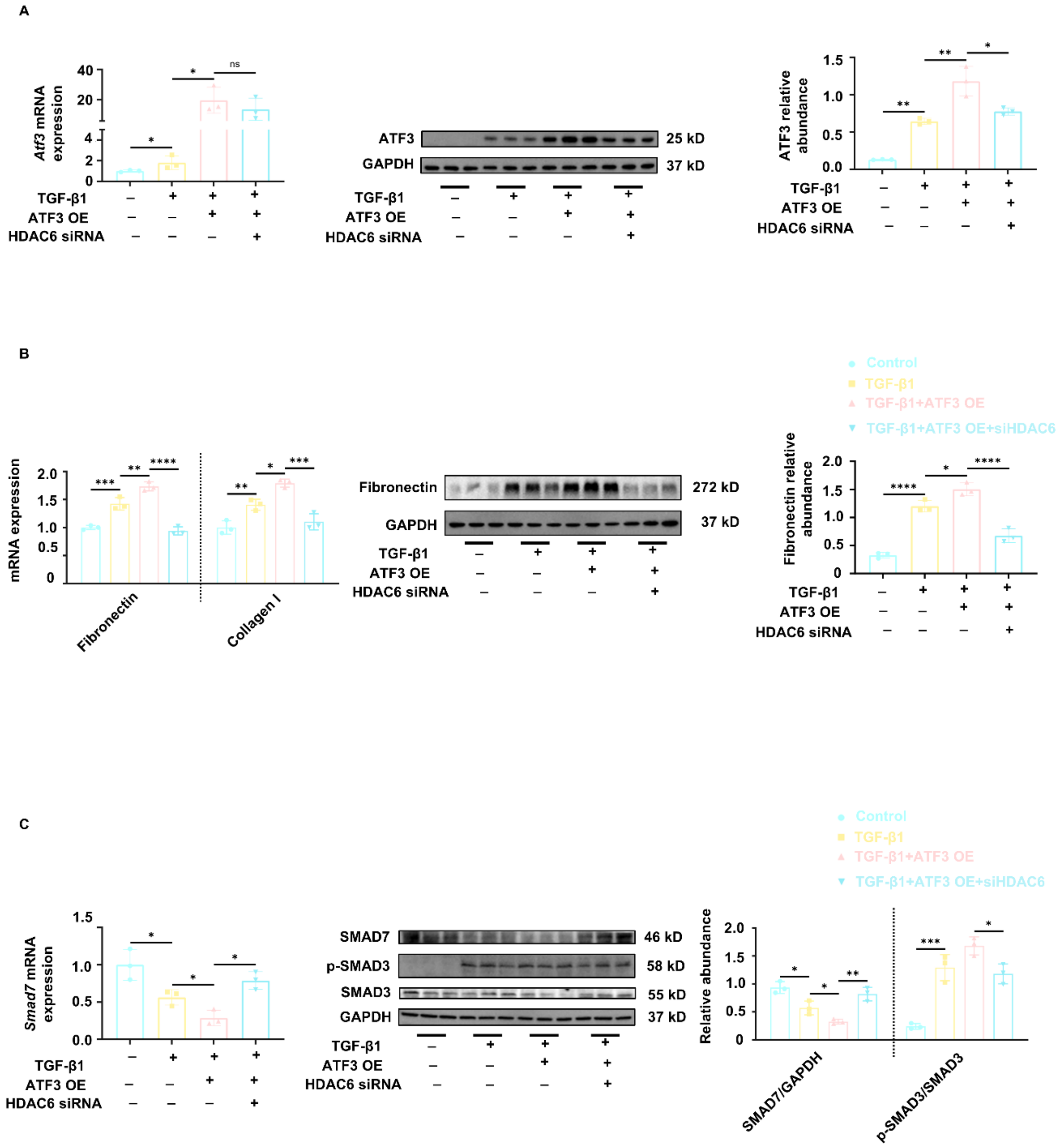
A
Atf3 mRNA expression
40
20
4
2
0
TGF-β1
ATF3 OE
HDAC6 siRNA
ns
ATF3
GAPDH
25 kD
37 kD
ATF3 relative abundance
1.5
1.0
0.5
0.0
B
Control
TGF-β1
TGF-β1+ATF3 OE
TGF-β1+ATF3 OE+siHDAC6
mRNA expression
2.0
1.5
1.0
0.5
0
Fibronectin
Collagen I
Fibronectin
GAPDH
272 kD
37 kD
Fibronectin relative abundance
C
Smad7 mRNA expression
SMAD7
p-SMAD3
SMAD3
GAPDH
46 kD
58 kD
55 kD
37 kD
Relative abundance
SMAD7/GAPDH
p-SMAD3/SMAD3

**Figure S9. ATF3 overexpression aggravated fibrosis in TGF-β1 treated 293T cells.** (A) Quantitative real-time PCR analysis and immunoblot of ATF3 in 293T cells. (B) Quantitative real-time PCR analysis and immunoblots of fibrotic markers in 293T cells. (C) Quantitative real-time PCR analysis of *SMAD7* and immunoblots of TGF-β/Smad3 signaling in 293T cells. *$p<0.05$, **$p<0.01$, ***$p<0.001$, ****$p<0.0001$.

A

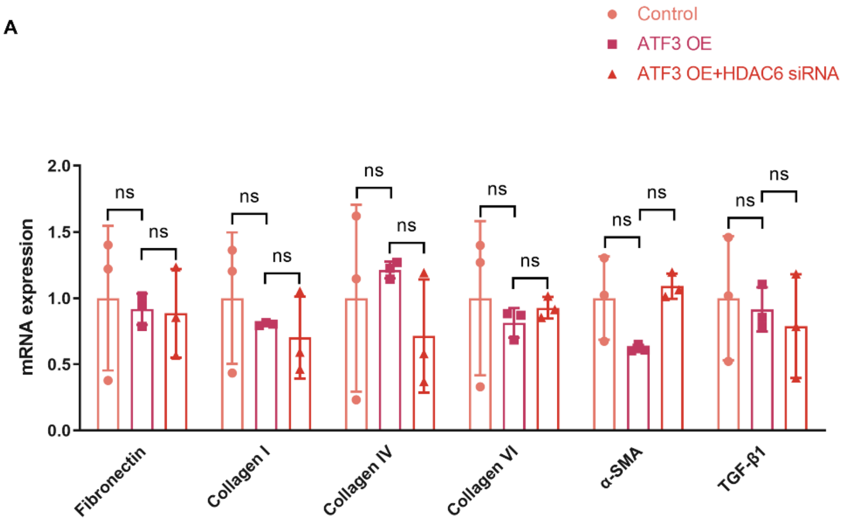

Control
ATF3 OE
ATF3 OE+HDAC6 siRNA
mRNA expression
2.0
1.5
1.0
0.5
0.0
ns
Fibronectin
Collagen I
Collagen IV
Collagen VI
α-SMA
TGF-β1


B

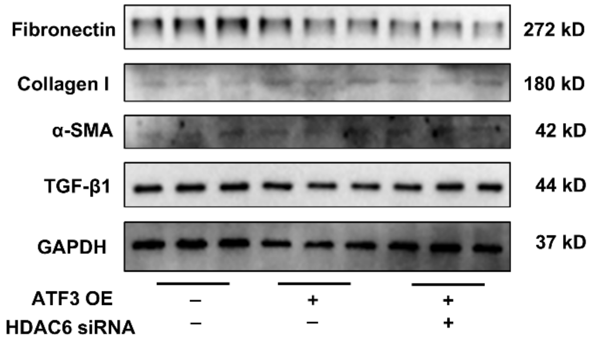

Fibronectin
272 kD
Collagen I
180 kD
α-SMA
42 kD
TGF-β1
44 kD
GAPDH
37 kD
ATF3 OE
–
+
+
HDAC6 siRNA
–
–
+


C

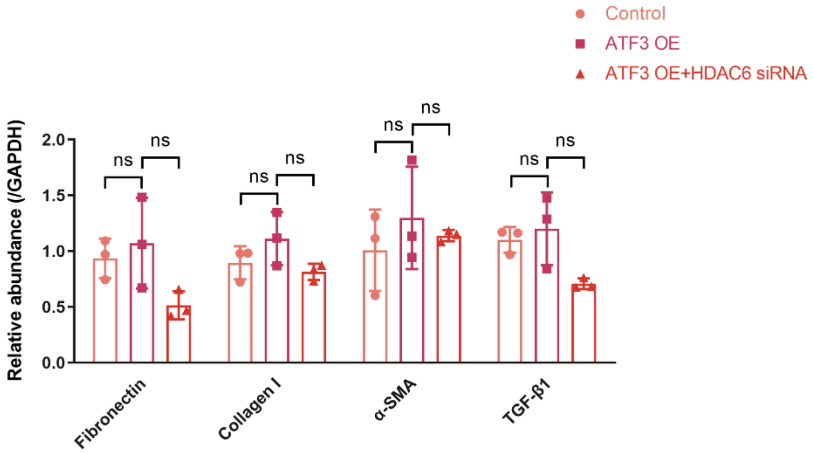

Control
ATF3 OE
ATF3 OE+HDAC6 siRNA
Relative abundance (/GAPDH)
2.0
1.5
1.0
0.5
0.0
ns
Fibronectin
Collagen I
α-SMA
TGF-β1

**Figure S10. The cell culture supernatants of TCMK-1 cells in each group stimulating NRK-49F cells could not affect fibrogenesis.** (A) Quantitative real-time PCR analysis, (B) immunoblots and (C) scorings of fibrotic markers in NRK-49F cells.

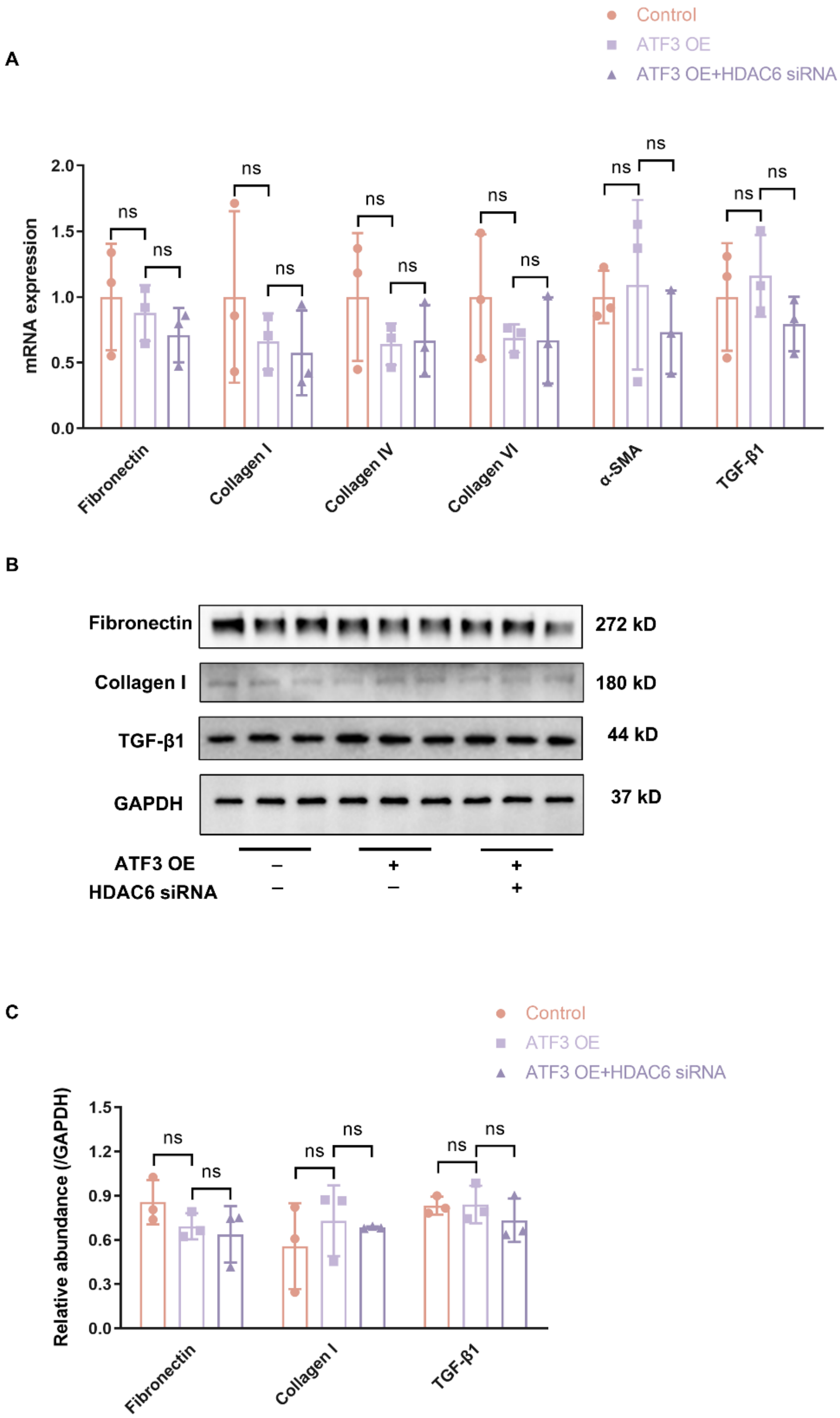
A
Control
ATF3 OE
ATF3 OE+HDAC6 siRNA
mRNA expression
2.0
1.5
1.0
0.5
0.0
ns
Fibronectin
Collagen I
Collagen IV
Collagen VI
α-SMA
TGF-β1
B
Fibronectin
272 kD
Collagen I
180 kD
TGF-β1
44 kD
GAPDH
37 kD
ATF3 OE
HDAC6 siRNA
C
Control
ATF3 OE
ATF3 OE+HDAC6 siRNA
Relative abundance (/GAPDH)
1.5
1.2
0.9
0.6
0.3
0.0
ns
Fibronectin
Collagen I
TGF-β1

**Figure S11. The cell culture supernatants of 293T cells in each group stimulating NRK-49F cells could not affect fibrogenesis.** (A) Quantitative real-time PCR analysis, (B) immunoblots and (C) scorings of fibrotic markers in NRK-49F cells.
